# Insights into Sinus-Lift Bone Grafting Materials: What’s Changed?

**DOI:** 10.3390/jfb16040133

**Published:** 2025-04-07

**Authors:** Anida-Maria Băbțan, Claudia N. Feurdean, Anca Ionel, Willi A. Uriciuc, Radu Chifor, Chambon Antoine Bernard Jaques, Bianca A. Boșca, Aranka Ilea

**Affiliations:** 1 IIIrd Department-Oral Rehabilitation, Faculty of Dentistry, “Iuliu Haţieganu” University of Medicine and Pharmacy, 400347 Cluj-Napoca, Romania; babtan.anida@umfcluj.ro (A.-M.B.); nicoleta.braitoru@umfcluj.ro (C.N.F.); ionel.anca@umfcluj.ro (A.I.); chifor.radu@umfcluj.ro (R.C.); aranka.ilea@umfcluj.ro (A.I.); 2 Faculty Nursing and Science for Health, “Iuliu Hațieganu” University of Medicine and Pharmacy, 400012 Cluj-Napoca, Romania; 3 Faculty of Dentistry, “Iuliu Haţieganu” University of Medicine and Pharmacy, 400347 Cluj-Napoca, Romania; 4 Ist Department-Histology, Faculty of Medicine, “Iuliu Haţieganu” University of Medicine and Pharmacy, 400347 Cluj-Napoca, Romania; bianca.bosca@umfcluj.ro

**Keywords:** sinus lift, bone graft, biomaterials, growth factors, bone regeneration

## Abstract

Background: Sinus-lift (SL) is a pre-prosthetic procedure with the objective of increasing bone height to achieve implant insertion primary stability in implant-supported prostheses. The biomechanical properties of SL augmentation materials are influenced by their origin, manufacture, bioactive substances addition, receiver, and surgical procedure. This systematic review provides insights into state-of-the-art SL biomaterials, focusing on autologous bone grafting as the gold standard. Methods: The study followed the PRISMA flow diagram, searching WoS (Web of Science), Embase, Cochrane, and PubMed databases using the search terms «sinus lift» OR «sinus augmentation» OR «bone graft» OR «bovine» OR «porcine» OR «autologous» OR «allogenic» OR «xenogeneic» OR «alloplastic» OR «hydroxyapatite» OR «β-tricalcium phosphate (β-TCP)» OR «equine» OR «PRF». Results: The highest bone gain was provided by Bioglass at 42%. Articles written between 2014 and 2024 in English or French, containing human studies and with full text available, were included. Participants were required to be in good general health, without acute, chronic, or congenital diseases, or substance abuse (drugs, alcohol, or nicotine). SL surgery was performed using the lateral approach, with no Schneiderian membrane perforation or postoperative complications. The network meta-analysis was conducted using the R statistical computing environment. To assess the inconsistency between direct and indirect evidence, we used a net heat plot. To evaluate heterogeneity across studies, we used the chi-squared-based Q-test and I^2^ statistic. A significance level of 0.05 was applied throughout all analyses. Results: Allogeneic bovine bone and hydrox yapatite demonstrated the lowest resorption rates. Significant differences were found for residual graft and connective tissue between allogenous bovine bone (ABB) + AlB vs. β-TCP + PRF (*p* = 0.028); ABB + AlB vs. β-TCP (*p* = 0.034); ABB + AlB vs. BCP (*p* = 0.037). Meta-analysis showed that the overall heterogeneity was 51.8% (6.9–75%; *p* = 0.019), with significant heterogeneity within designs (*p* = 0.007) and no significant heterogeneity between designs (*p* = 0.39). AB had a better bone regeneration ratio compared to many of the other interventions, but only two passed the threshold of significance: A1B and B-TCP + AB. Conclusions: A grafting material’s superiority is determined by its new bone formation ratio, connective tissue integration, residual graft content, and bone resorptionratio. Although autologous bone grafting has exhibited superior bone regeneration compared to other biomaterials, it was not favored due to its unpredictable connective tissue concentration and bone resorption ratio. Additionally, autologous bone exhibited the fastest metabolic turnover among all grafting materials.

## 1. Introduction

Prolonged maxillary edentulous status leads to alveolar ridge resorption, which is further exacerbated by maxillary sinus pneumatization, contributing to progressive bone volume loss. To address this issue, clinicians have developed surgical techniques aiming to restore adequate bone volume. Sinus lift (SL) was described for the first time by Dr. Hilt Tatum in 1976 and published in 1980 by Dr. PJ Bone and RA James [1]. SL is a surgical procedure that augments bone volume at the sinus floor, facilitating immediate or delayed implant placement. Volume is achieved using different types of grafting material, from autogenous bone to bone substitutes, each one possessing unique individual mechanical and biological properties and resorption ratios.

SL’s main clinical indications are insufficient bone height in the upper premolar and molar area and insufficient bone density (which might compromise implant primary stability). It can also be a surgical procedure used in oro-antral communication closure, for the management of palatal clefts, or as an interposed bone graft in Le Fort type I fracture [2,3]. Despite its benefits, the SL procedure is invasive and requires specific postoperative management. Contraindications of this technique are related to historical oncological radio/chemotherapy in the cephalic extremity, unbalanced systemic diseases (autoimmune, diabetes, cardiovascular), acute maxillary sinusitis, acute rhinitis, bad oral hygiene, chronic smoking, alcoholism, or psychiatric disorders [4]. These limitations can be overcome by appropriate medical intervention, treatment of the acute stage, and stabilization of psychological status before oral surgery.

SL bone augmentation techniques

There are two main techniques for SL bone augmentation: the direct technique, with a lateral approach, and the indirect technique, with an osteotomes and crestal approach. The SL lateral procedure has been demonstrated by Tatum, describing the osteotomy in the lateral maxillary wall needed to create a window to visualize and elevate the sinus Schneider membrane [5]. The limits of the osteotomy are driven by the number of needed implants and the position of the posterosuperior artery. The detached bone fragment can be used as a graft or as the new maxillary floor. If there is a 3–4 mm residual height, immediate implant placement might be approached. The direct technique is advantageous due to the high bone volume that might be achieved. However, it has a risk of perioperative morbidity and Schneider membrane perforation.

In the indirect technique, the sinus membrane is elevated transalveolar, meaning that a bone fragment adjoining the sinus membrane is elevated. Tatum first described this method, and then Summers demonstrated a different crestal approach with sequential crestal osteotomies to achieve sinus membrane elevation [5]. The indirect approach is indicated where the residual bone is equal to or higher than 6 mm, as it enables immediate implant placement, lower inflammatory manifestation, and short surgery procedure duration. The most common intraoperative complication is membrane perforation during elevation, osteotomy, or grafting material excess; small perforations (5–10 mm) can be managed by applying a resorbable collagen membrane, whereas larger perforations (>10 mm) require slow-resorbing reticular membranes. If left untreated, these accidents could lead to acute/chronic maxillary sinusitis, bleeding, oro-antral fistula, and sinus graft discharging [6]. Another potential complication is superoposterior artery and infraorbital nerve injury, which might be mitigated by a proper preoperative tridimensional approach, using piezo surgery instead of rotative instruments, limiting the upper board of the bone window, given the fact that that the distance between the alveolar ridge and artery is approximately 18 mm [7].

SL postsurgical complications

Immediate SL complications (within the first 24 postoperative hours) include hemosinus—draining through the nasal fossa or the suture knots—pain, and facial edema [8]. Late complications are less frequent but are challenging to manage. Membrane perforation might result in sinus graft particle expulsion, which can obstruct ostium drainage and cause reactive sinusitis. Additionally, the surplus of grafting material can surpass the osteotomy window and leak into the oral cavity, affecting mucosal healing. Eighth-day acute sinusitis onset occurs in anaerobic preoperatory contaminations/over-infection caused by insufficient ostium drainage and can be cured quickly in the physiologically sound sinus membrane. Third-week sinusitis—chronic—is three times more frequent and is caused by numerous factors that will lead to impaired ostium drainage or acute non-treated sinusitis [9]. After SL healing time, cystic transformations might appear: mucocele as a consequence of maxillary secretion and ostium dysfunction, leading to a pseudotumoral evolution and associated bone resorption; it is essential to differentiate this mucocele by mucosal cysts—the consequence of non-secretory, glandular cells, which have a radiolucent, stationary appearance on imaging [8]. Another potential complication, implant migration, can occur caused by a lack of primary stability and nasal and intrasinus pressure differences. Graft rejection may emerge immediately or years post-SL. Detachment of the grafted material can be triggered by an acute sneezing episode, hard nutriment mastication, or postoperative sinus trauma [9].

The success of SL surgery depends on several anatomical characteristics of sinus cavities and their communication with the nasal fossa via the ostium and, equally importantly, on the features of the grafting materials and membranes. The most important of these is osteoconduction—defined as the material passive characteristics for achieving bone regeneration, which allows cellular and vascular invasion of the surrounding tissues [10,11]. Implant integration, prior to the prosthetic stage, is a biological phenomenon that can only take place in well-vascularized, physiologically dynamic cellular live bone tissue. The bone metabolism, governed by osteoclastic and osteoblastic cycles, enables the transformation of the graft material into physiological bone. The second property is osteoinduction—the capacity of autonomous chemical substances to recruit mesenchymal cells and differentiate them into pre-osteoblasts and osteoblasts, capable of synthesizing the mineralizing bone matrix. Among the most potent osteoinductive agents are the chemical agents—cytokines, growth factors, and the famous bone morphogenic proteins (BMPs), which have the ability to induce bone neoformation in receptor sites. Despite the advancement in allogenic materials and bone morphogenetic protein-2 (BMP-2), the only graft that has this property is the autogenous graft [12]. Osteogenesis refers to the direct neoformation of bone from osteogenic cells, the autogenous graft being the only osteogenic material (it has live osteocytes), unlike allogenous and xenogenous material, that needs to be pretreated before becoming acellular grafts, minimizing the risk of viral/bacterial cross-infection [12].

**Figure 1:**
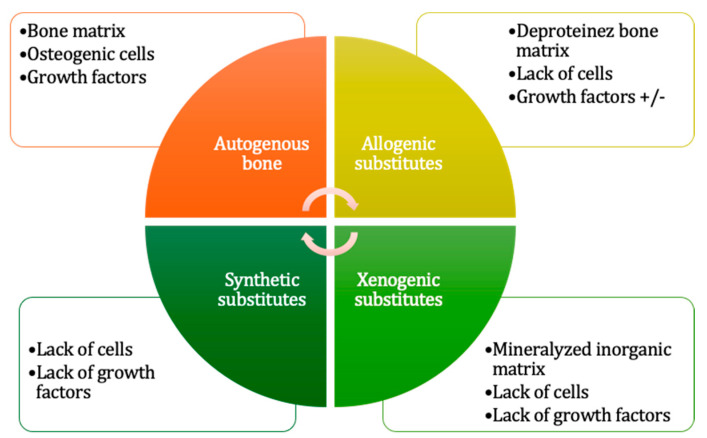
SL grafting materials.

The following section provides a concise overview of different grafting materials (Figure 1) for a better understanding of this paper’s objective. While all four categories exhibit osteoconductive properties, only the autogenous graft has all three essential properties—osteoconduction, osteoinduction, and osteogenesis. Allogenic substitutes, by contrast, allogenic substitutes are osteoconductive and osteoinductive but lack intrinsic osteogenic capacity.

Grafting materials

1. Autogenous grafts

Autogenous grafts are considered the “gold standard” due to their superior properties. Autogenous refers to the self-harvesting site, which depends on the amount of bone volume needed. The advantages are numerous—excellent biocompatibility, absence of viral transmission risks, lack of immunogenic response, and high mesenchymal osteogenic response. However, the disadvantages include surgery morbidity, which is due to the necessity of a second surgical situs for graft harvesting with consequent limited bone availability and an unpredictable resorption ratio [11]. Harvesting procedures for both cortical and trabecular grafts include intra- and extra-oral sites. Intra-oral sites are beneficial (less morbidity and faster healing time) and are represented by the mental and retromolar regions; extra-oral sites include the parietal bone and iliac crest. All harvesting procedures require careful attention regarding nervous and vascular structures. Injury to the incisal nerve would lead to labio-mental hypoesthesia, while damaging the inferior alveolar nerve would lead to lower lip anesthesia. Additionally, submental or facial artery injury can result in a hemorrhage that is not easy to manage [11].

2. Allografts

Allografts are obtained from either living or recently deceased (no more than 24 h postmortem) human donors. The grafts are treated, sterilized, and conditioned before being stored in bone graft banks for distribution. The allografts can be conditioned under blocks, cortico-trabecular powder, gel, or paste (after demineralization). Graft banks have been successfully used for more than 50 years in orthopedic surgery and have been used in dentistry since the 1970s (Olivier SAAD 2018) [12]. Allografts are largely used in the United States, and Europe has also started to use them. While no cases of cross-infection have been reported, there remains a theoretical risk of non-identified pathogen transmission. Harvesting is contraindicated in patients diagnosed with human immunodeficiency viruses (HIV) 1 and 2, hepatitis B and C, syphilis, and T-lymphotrope human HTLV-1 virus [13].

There are different types of allografts depending on the manufacturing process. Freeze-dried bone allograft (FDBA) undergoes chemical treatment in order to inactivate virus and pathogen elimination and is subsequently lyophilized (a preserving process that associates voids and low temperatures to dehydrate the graft without altering it). One consequential advantage is that it can be stored at room temperature for up to five years, simplifying transport and storing logistics. Demineralized freeze-dried bone allograft (DFDBA) undergoes an additional demineralization process to remove the mineral bone matrix, allowing it to be marketed as paste, soft layers, and gels in syringes. Another allograft type is the deproteinized-delipidized type, which is not lyophilized; as a consequence, it does not lose its mechanical properties, such as its strength against deformation and its pressure, which is comparable to that of natural bone [13].

3. Bone substitutes

Animal substitutes (xenogenic) have various origins: bovine, ovine, porcine, and equine. Bovine grafts are highly favored due to their structural and compositional similarity to human bone, allowing good vascular cell migration, osteoconduction, and osteointegration. Xenogenic grafts are advantageous because of their availability; they come in the form of powder, a powder and collagen matrix, blocks, gels, and putty. However, patient reluctance due to personal/religious beliefs, misinterpretation, or misinformation may limit their use [14].

Choral substitutes are derived from marine choral exoskeleton, a porous structure of calcium carbonate that is comparable to human trabecular bone; this can be transformed into hydroxyapatite (HA) using high-temperature sintering. Despite its high compression strength, choral grafts are brittle and have a lower osteoconduction capacity than other substitutes. It has been shown that its long-term resorption ratio is low, which allows volume stability, potentially hindering bone remodeling [14].

Alloplastic synthetic substitutes have no biologically derived tissue and comprise 60% of the bone substitutes market. They pose no viral transmission risks and are suitable for ethnic/religious restrictions; common examples include phosphor-calcic ceramics—HA—which is dense or porous, with a 100% survival ratio but lower vital bone [15]. β-tricalcium phosphate (β-TCP) and alpha (α-TCP) are osteoconductive substitutes that are more soluble than HA, allowing mineralized new bone to inhabit. There are also mixtures of HA 60–40% β-TCP and bioactive bone, which is a full-resorbable material with osteoconductive and osteoinductive properties that allow bone formation at the particles’ contact and a distance from bone tissue; it is physicochemically linked and will release important ions such as sodium, calcium, phosphor, and silicium, which will enhance osteoblast proliferation. Iit can be mixed with drugs, proteins, and growth factors [16].

4. Growth adjuvants

Platelet-rich-plasma (PRP) is a blend of a high concentration of growth factors, including platelet-derived growth factors, transforming growth factor beta (TGFβ), insulin-like growth factor (IGF), epidermal growth factor (EGF), and vascular endothelial growth factor (VEGF). PRP is obtained via centrifugation of the patient’s blood for 10 min at 2400 rpm with a second cycle of 15 min at 1600 rpm after removing leucocytes and erythrocytes. When combined with bone substitutes, PRP enables higher maturation and bone density [17]. Platelet-rich-fibrin (PRF) is a fibrin clot containing numerous platelets, leucocytes, and growth factors, obtained by centrifugation with a single cycle of 10 min at 3000 rpm. PRF can be mixed with grafting material or flattened and used as a covering membrane to protect bone grafts [18].

5. Synthetic membranes

SL is a guided bone regeneration (GBR) procedure, meaning that the bone graft needs to be covered with a membrane, preventing epithelial cell migration into the defect area, favoring colonization of the situs with cells capable of synthesizing new bone to stabilize the flap and the graft, and to provide osteogenesis space. Depending on their degradation ratio, GBR uses two types of membranes: non-resorbable and resorbable. Non-resorbable Polytetrafluoroethylene (PTFE) is manufactured in three different types: expanded (e-PTFE), which is an elastic membrane with one soft side that leaves growing space and does not allow fibrous tissue development and a porous thick surface that will inhibit epithelial growth. e-PTFE comes with the disadvantage of exposure risk and bacterial graft contamination, a risk which is overcome by dense (d-PTFE), which has high porosity and thickness and does not need flap closure, itself being sufficient for graft protection; this property makes it favorable for use with extended GBR defects [19]. Titanium mesh membranes are another option, offering stability and adaptability. However, they can be defect-shaped, their disadvantages being the exposure risk (which might induce microbial contamination) and the requirement of a second surgery for their removal [20]. The second type is the resorbable membranes used in SL, which do not require removal. However, their degradation ratio is highly unpredictable, which might affect the quality of the regenerated bone. They are also often difficult to suture, and might collapse, leading to graft dispersion. Resorbable membranes include collagen, pericardium, PRF, acellular dermal matrix, and xenogenic membrane, which are widely used in implant procedures [20].

Grafting procedure evaluation

The bone graft evaluation gold standard is histological qualitative and quantitative analyses of a regenerated bone block harvested with a trephine burr. Subsequently, an analysis of bone density is performed to evaluate the percentage of osteocyte-graft material contact, lacunar areas of fibrous tissue formation, inflammatory process detection, necrosis, and osteoblast migration to the grafting material area. The quantitative analysis identifies residual material, specific inflammatory cells and vessels, osteogenesis, and osteolysis [21]. Radiological tridimensional assessment (cone beam computer tomograph—CBCT) provides a high-resolution view of the graft; if no implant is present, the surgeon evaluates the height (Misch classification) and density (Hounsfield units) while detecting the active resorption process. If an implant is inserted, osteointegration can be assessed using the contact between the whorls and regenerated bone. Good osteointegration is represented by the continuity of the bone–implant contact along its length [22].

The implant is more stable as the receptor bone has a thicker cortical area. Implant stability and monitoring are available for insertion torque (IT) and resonance frequency (RF). IT quantifies the force needed for implant insertion, indicating intimate contact between the bone walls and the implant surface. IT can only be measured at the moment of insertion and is non-repeatable [23]. RF is calculated according to the implant’s response signal to soundwaves, giving the implant’s stability, or implant stability quotient (ISQ), on a scale ranging from 1 to 100. This evaluation is repeatable at specific times (postoperatively, during the healing period, before inserting the healing caps, before prosthetic screwing, or at annual check-ups) to verify if the stability is constant or decreased [24]. Implant survival is of great research interest and is defined as the presence of an implant and its prosthetic rehabilitation in the patient’s oral cavity after a time period established by the scientist. Implant survival is expressed as a percentage and allows survival comparisons of each technique, graft reliability, implant type, and specific comorbidities [25], evaluating the long-term success of the surgical procedure and gauging the quality of implant and graft manufacturers. With all these surgical possibilities, it is important that the surgeon knows the advantages, disadvantages, and limits of each. Numerous studies have been conducted to verify these hypotheses.

The purpose of this systematic review is to compare clinical, histological, and radiological bone grafts and graft substitutes, whether they should be aggregated with growth factors or not, how they are used in healthy patients to refine the autologous bone graft gold standard, and whether an allograft or a material mixture might replace them.

## 2. Materials and Methods

**Figure 2:**
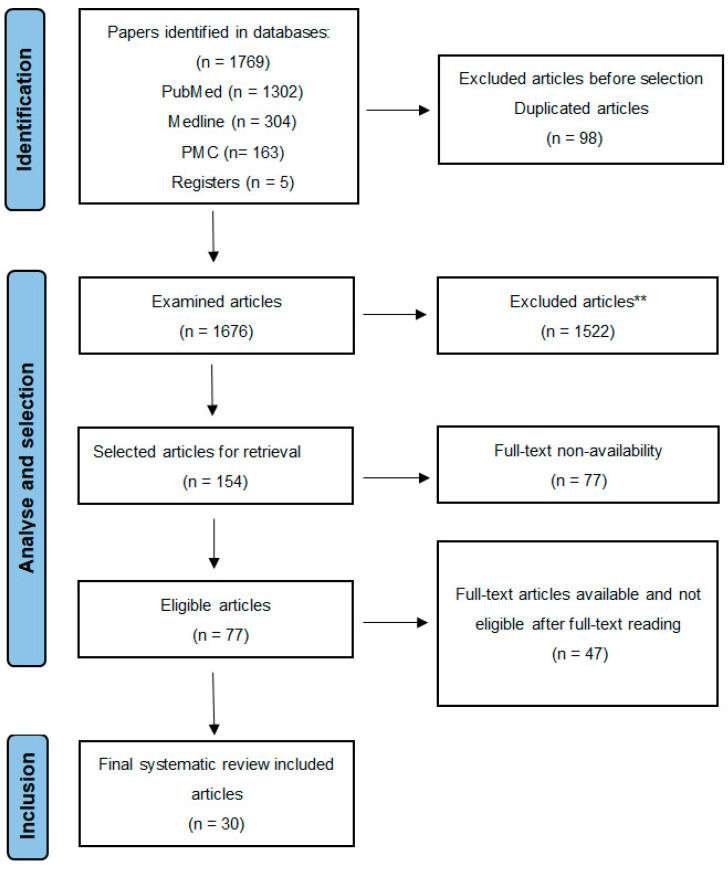
Articles’ preselection and selection PRISMA chart; ** Articles excluded due to irrelevance for the research question and insufficient data.

This systematic review was conducted following the Preferred Reporting Items for Systematic Reviews and Meta-Analyses flow diagram (PRISMA chart). The research databases utilized included Embase, Web of Science (WoS), Cochrane, and PubMed. The keywords used for the search were: «Sinus lift» OR «sinus augmentation» AND «bone graft» OR «bovine» OR «porcine» OR «autologous» OR «allogenic» OR «xenogeneic» OR «alloplastic» OR «hydroxyapatite» OR «β-tricalcium phosphate» OR «equine». Studies published between 2014 and 2024 were considered for inclusion. The inclusion criteria encompassed full-text articles written in English or French within the specified timeframe. Eligible studies involved human participants—patients were in good health, without any acute, chronic, or congenital disease, and non-addicted to drugs, alcohol, or nicotine. SL surgery was to be performed using a lateral approach without Schneider membrane perforation or postoperative complications. In the initial stage of the selection process, three different reviewers individually assessed titles, abstracts, and then the full texts to minimize the potential risk of bias. Texts of the studies that met all the inclusion criteria were independently read and again checked for eligibility. At the final stage regarding PRISMA chart evaluation and inclusion, a discussion was conducted to ultimately decide on the inclusion or exclusion of the selected studies and to proceed to data extraction. At each stage of the analysis, selection, and inclusion, any remaining discrepancies were resolved by a third supervisor for arbitration. After applying the PRISMA flow diagram (Figure 2) and inclusion and exclusion criteria, 30 articles were included in the systematic review.

The primary evaluated parameter was new bone formation, assessed in relation to the type of graft material used after a six-month healing period. Bone regeneration ratio is the histomorphological analysis goal, obtained from qualitative and quantitative information of the biopsy bulk regarding bone remodeling, medullar space composition, connective tissue, and residual graft particles that were not integrated into the osteogenesis process. Histological images were processed, and the percentages or surface results are given. The bone resorption ratio was determined by volumetric comparison analysis using CBCT scans, comparing preoperative images with the ones made six months post-SL procedure. Tridimensional reconstruction allows bone graft visualization and volume difference determination between the two points, expressed in cm^3^, mm^3,^ or percentages. Fischer-based ANOVA tests were used for statistical analysis.

Statistical analyses

The network meta-analysis was conducted using the R statistical computing environment (R Foundation for Statistical Computing, Vienna, Austria), specifically version 4.1.2, in combination with the netmeta package. Given the potential clinical heterogeneity among trials, we applied the restricted maximum likelihood (REML) method to estimate the parameters of the random-effects model. The analysis generated mean differences with corresponding 95% confidence intervals. A frequentist approach was employed for the network meta-analysis, with AB designated as the reference treatment.

The study first illustrated the structure of treatment comparisons through network graphs. We then analyzed and visualized the contribution of direct and indirect evidence for each comparison. Additionally, mean path length and minimal parallelism statistics were computed to enhance interpretability.

Both the pooled effect estimates from direct evidence alone and those derived from combined direct and indirect evidence were calculated for all comparisons and were summarized in a network league table. The primary treatment comparisons, using AB as a reference, were illustrated in forest plots. The ranking of treatment effects was determined using the P-score frequentist method. To evaluate potential inconsistencies between direct and indirect evidence, we used a net heat plot. To evaluate heterogeneity across studies, we used the chi-squared-based Q-test and I^2^ statistic. A statistical significance threshold of 0.05 was applied to all analyses.

## 3. Results

### 3.1. SL New Bone Regeneration Ratio

From the included studies, 22 articles analyzed SL new bone formation ratio, including 18 different graft types, multiple graft mixture, or growth factors. We found nine different single graft samples: autogenous bone (AB), allogenic bone (AlB), inorganic bovine xenogenic bone (IBB), equine xenogenic bone (EB), beta tricalcium phosphate (β-TCP), biphasic calcium phosphate at different concentrations (BCP), hydroxyapatite (HA), bioapatite collagen (BC), and bioactive glass ceramic (Bioglass). We finally had a total of nine different samples of graft or graft-adjuvant mixtures: beta tricalcium phosphate + autogenous 1:1 (β-TCP + AB), inorganic bovine xenogenic bone + autogenous 1:1 (IBB + AB), allogenic + autogenous 1:1 (AlB + AB), bioactive glass ceramic + autogenous 1:1 (bioglass + AB), allogenic + inorganic bovine xenogenic bone 1:1 (AlB + IBB), inorganic bovine xenogenic bone + platelet-rich fibrin (ABB + PRF), biphasic calcium phosphate + platelet-rich fibrin (BCP + PRF), hydroxyapatite + platelet-rich fibrin (HA + PRF), and inorganic bovine xenogenic bone + bone marrow concentrate (ABB + BMC). All values recorded are reported in Table 1, and Supplementary S1.

The averages obtained for the new bone regeneration ratio were, respectively: AB = 40.4%; AlB = 22.9%; IBB = 26.5%; EB = 22.4%; β-TCP = 38.2%; BCP = 28.3%; HA = 31.5%; BC = 21.4%; Bioglass = 42.0%; β-TCP + AB = 31.3%; IBB + AB = 36.6%; AlB + AB = 41.0%; Bioglass + AB = 39.5%; AlB + IBB = 24.4%; IBB + PRF = 27.4%; BCP + PRF = 18.6%; HA + PRF = 30.3%; IBB + BMC = 30.3%.

**Figure 3:**
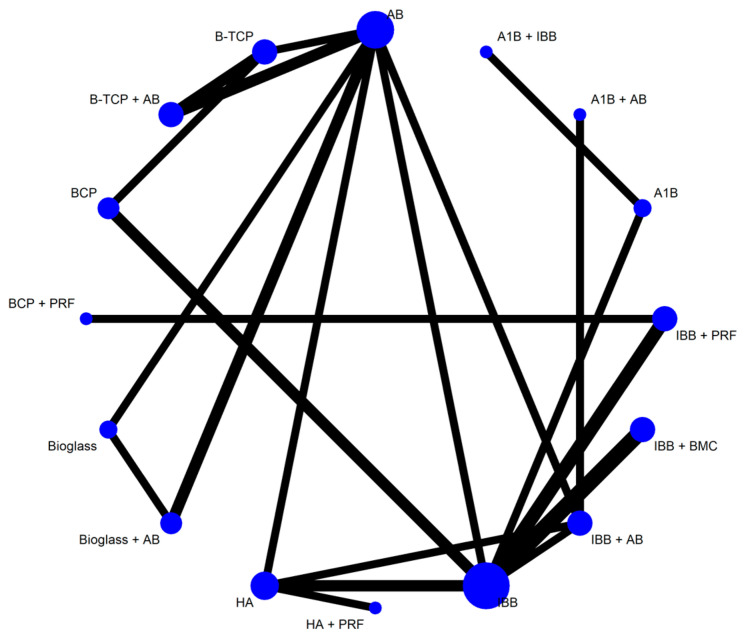
Network plot for sinus lift new bone regeneration ratio after six months of healing time.

A network meta-analysis was performed on the 22 studies presented in Table 1. This comprised 21 pairwise comparisons of 16 treatments. The overall heterogeneity was 51.8% (6.9–75%), *p* = 0.019, with a significant heterogeneity within designs (*p* = 0.007) and no significant heterogeneity between designs (*p* = 0.39). The network plot is presented in Figure 3.

**Figure 4:**
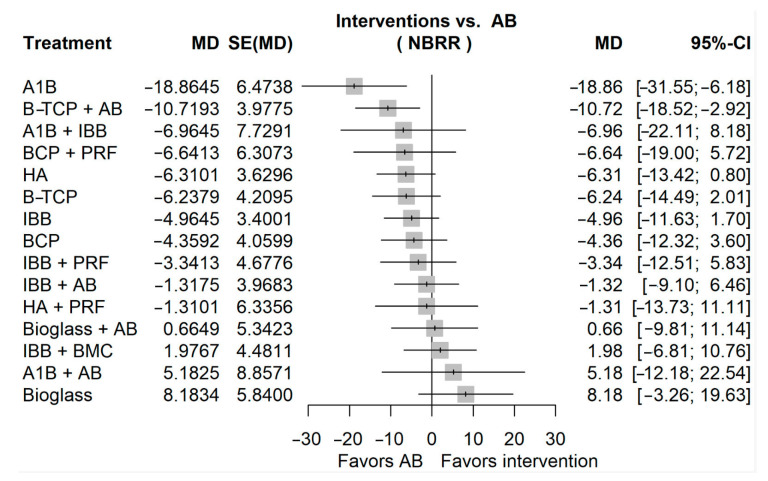
Forest plot for sinus lift new bone regeneration ratio after six months of healing time. (MD, mean difference; SE, standard error; AB, autologous bone graft; CI, confidence interval; NBRR, new bone regeneration ratio).

The direct and indirect evidence for sinus lift new bone regeneration ratio after six months of healing time is presented in Supplementary Figure S1. A few comparisons have a high proportion of direct evidence (especially regarding IBB), indicating that they are well-supported by direct studies. Many comparisons rely on indirect evidence, suggesting that direct head-to-head comparisons are scarce. A few comparisons have a high parallelism, indicating improved reliability. Some comparisons have low parallelism, indicating that they are highly dependent on indirect evidence. Some treatment comparisons require multiple indirect links (longer path length), reducing the strength of the inference. The most reliable comparisons are those colored in orange at the beginning of the plot. The league table containing all the comparisons between all the treatments is presented in Supplementary Table S1. The main result, represented by the network meta-analysis forest plot, is presented in Figure 4. The AB was selected as a reference group. AB had a better bone regeneration ratio compared to many of the other interventions, but only two passed the threshold of significance: A1B and B-TCP + AB. Bioglass had a higher bone regeneration ratio compared to AB but without passing the threshold of significance. The previous order of the treatment rankings can be observed in Supplementary Table S2.

### 3.2. Connective Tissue Quantity and Residual Graft

To evaluate connective tissue and residual graft, nine different graft samples were used: AB, AlB; IBB, inorganic porcine bone (IPB), BCP, β-TCP, HA, EB, and Bioglass. Five graft mixtures or graft-adjuvant samples were investigated: ABB + AB, AlB + AB, ABB + AlB, β-TCP + PRF, and ABB + FRP. All the values recorded are presented in Table 2, and also described in Supplementary S2.

**Figure 5:**
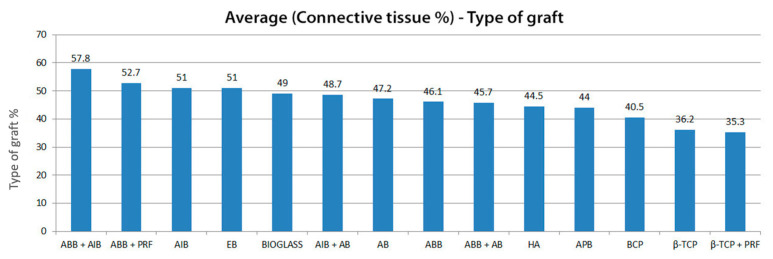
The average quantity of connecting tissues with their 95% confidence intervals, depending on the type of graft.

**Figure 6:**
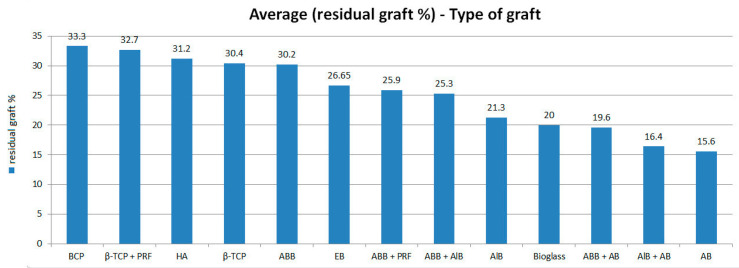
The means of residual graft quantity with their 95% confidence intervals, depending on the type of graft.

**Figure 7:**
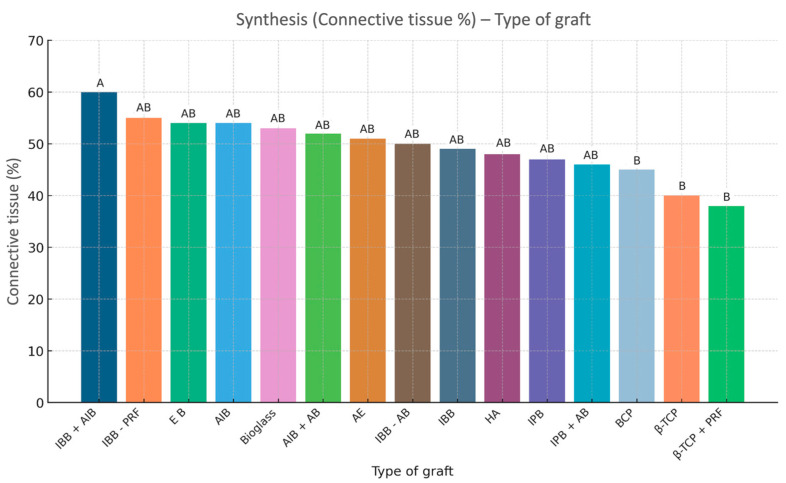
Summary of comparison of connective tissue quantity averages. Each variable with at least one letter in common (A or B) is not statistically significant.

**Figure 8:**
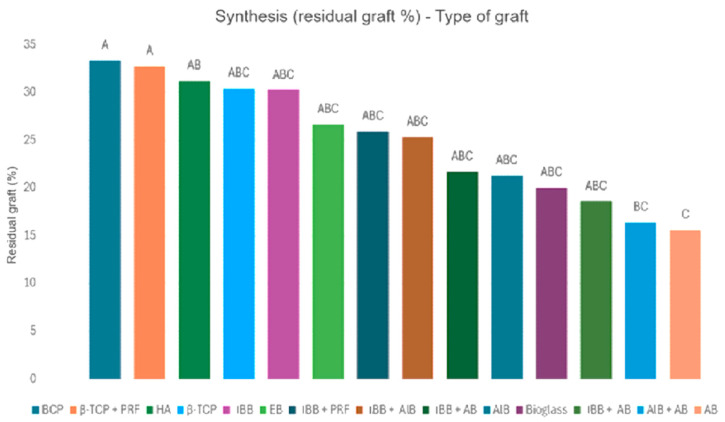
Summary of comparison of the average quantity of residual graft. Each variable with at least one letter in common (A or B or C) is not statistically significant.

The averages obtained for the quantity of connective tissue were, respectively: ABB + AlB = 57.8%; ABB + PRF = 52.7%; AlB = 51.0%; EB = 51.0%; Bioglass = 49.0%; AlB + AB = 48.7%; AB = 47.2%; ABB = 46.1%; ABB + AB = 45.7%; HA = 44.5%; APB = 44.0%; BCP = 40.5%; β-TCP = 36.2% and β-TCP + PRF = 35.3%. These averages are visualized with their 95% confidence intervals in Figure 5. As for the averages on the residual quantity of graft, the results obtained are: BCP = 33.3%; β-TCP + PRF = 32.7%; HA = 31.2%; β-TCP = 30.4%; ABB = 30.2%; EB = 26.65%; ABB + PRF = 25.9%; ABB + AlB = 25.3%; AlB = 21.3%; Bioglass = 20.0%; ABB + AB = 19.6%; AlB + AB = 16.4% and AB = 15.6%. There are no data on the residual graft quantity for the porcine xenogenic graft (IPB). These averages are visualized with their 95% confidence intervals in Figure 6. Also, the summaries regarding the comparison of connective tissue quantity averages and the average quantity of residual graft are graphically visualized in Figure 7 (statistical significance for connective tissue) and Figure 8 (statistical significance for the amount of residual graft).

### 3.3. Bone Resorption Ratio

We investigated six different biomaterial samples—AB, AlB, IBB, β-TCP, HA, and Bioglass—but also four samples of different grafting mixtures in 1:1: IBB + AB; β-TCP + AB; Bioglass + AB; IBB + AlB. All the values recorded are presented in Table 3, and also included in Supplementary S3.

**Figure 9:**
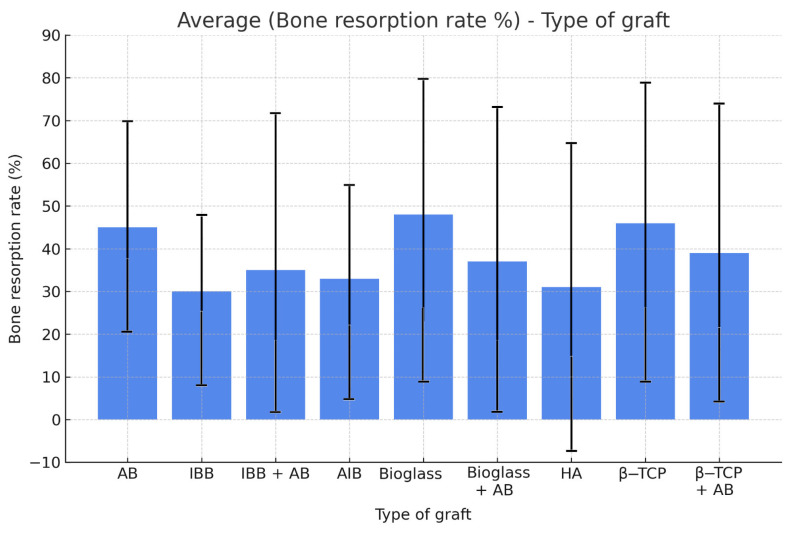
Histogram graph of bone resorption averages with their 95% confidence intervals, depending on the type of graft.

**Figure 10:**
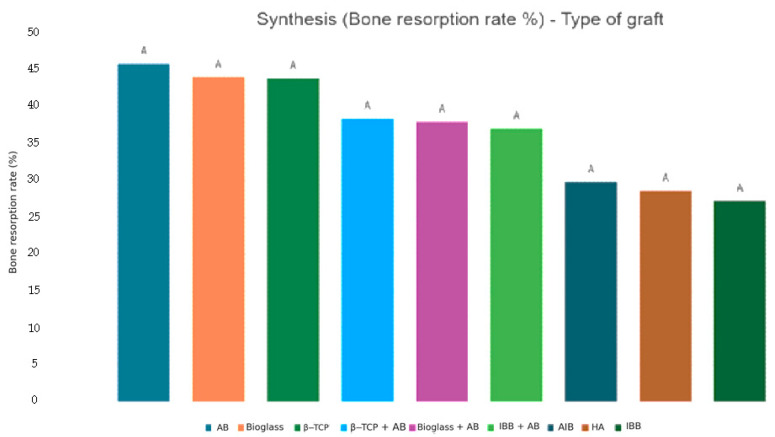
Summary of comparison of bone resorption averages. Each variable with at least one letter in common (A) is not statistically significant.

The averages obtained for bone resorption are, respectively: AB = 45.7%; AlB = 28.3%; IBB = 27.2%; β-TCP = 43.8%; HA = 28.5%; Bioglass = 44.0%; IBB + AB = 37.0; β-TCP + AB = 38.3%; Bioglass + AB = 37.9%; and IBB + AlB = 12.6%. These averages are displayed with their 95% confidence intervals in Figure 9. The Fischer-based ANOVA analysis revealed no significant difference between these different samples. The *p*-value of the two extremes, AB vs. IBB, was 0.184 > 0.05. The significant comparisons and differences can be seen in Figure 10. All comparisons and *p*-values can be found in Supplementary S4.

## 4. Discussions

Regarding the gain of new bone, the extreme maximum or minimum averages, such as Bioglass, AlB + AB, BC, and, in particular, BCP + PRF, come from values from a single 2016 study by Taschieri et al., with a sample size of only 10 individuals [29]. Given the limited sample, it is therefore difficult to consider these values as fully representative of bovine bone grafting. If we exclude the average of BCP + PRF, there would be no emerging significant difference between the samples, which would suggest that there is no difference in the osteogenesis reaction between the different types of grafts, which would challenge the hypothesis that autogenous grafting remains the gold standard for sinus lift surgery. However, it is worth noting that the average gain of new bone per autogenous graft is among the highest, lower only than Bioglass and AlB + AB. Furthermore, we note that the bone gain averages of mixtures of different types of graft with AB in a 1:1 mixture are also in the upper half of the averages and often higher than the average of the same type of graft without a mixture including AB. Finally, we can also assume that the presence of adjuvants such as PRF or BMP does not significantly impact bone formation compared to the same type of graft without adjuvant.

Regarding the amount of connecting tissue, the highest recorded average—for IBB + AlB—is based on a single study and observation. Furthermore, the sample size is not specified in the study by Danesh-Sadi et al. [53]. It is therefore difficult to recognize this value as being representative of this type of grafting. When excluding this data point, there is no longer a significant difference between our means, suggesting that there is no difference in bone architecture between the different graft types, and they yield similar amounts of connecting tissue, marrow space, and, therefore, vascularization. Consequently, this would mean that autogenic transplantation cannot be considered the gold standard in SL surgery, as its performance aligns with the overall mean. We can nevertheless note that the lower averages are those coming from alloplastic-type grafts, which may suggest that this type of graft has a less effective vascular and connective architecture than the others.

Compared to the residual graft quantity results, we note that AB differs significantly from BCP, β-TCP + PRF, and HA. AB has the lowest average of all the techniques, which would indicate more complete metabolism by the human body and faster osteogenesis for the autograft. However, it is not significantly different from the majority of other types of graft, which leads us to conclude that AB cannot be considered the gold standard at this level either. Notably, alloplastic-type grafts exhibit the highest residual graft values, suggesting that their metabolism by the human body and osteogenesis are slower and less complete.

With respect to bone resorption, no significant differences were observed among grafting materials, indicating that the different types of grafting materials have similar osteoclastic activity. Therefore, there does not seem to be any difference in the osteoclast activity and metabolism of the graft by the receptor between the different biomaterials or mixtures of biomaterials. GBR objectives are for bone resorption to be minimal and to allow maximum bone to be obtained for implant placement. Since AB does not differ significantly from the rest of the grafting materials, it cannot be considered the gold standard in SL surgery. Interestingly, we observe that AB exhibits the highest average bone resorption; this warrants further studies with larger samples to verify this hypothesis.

To compare and validate these findings, we examined several systematic reviews, including the study by Trimmel et al. [56] on the gain of new bone from different biomaterials. It brings together 35 studies over a period of 16 years (2003–2019). Out of 378 different comparisons, only two are significant: IBB + BMC vs. biodegradable copolymer (BC) and ABB + BMC vs. AlB, with IBB + BMC having superior results. The remaining 376 comparisons are not significant, reinforcing our conclusion that AB is no different from the other grafts, demonstrating that it cannot be considered the most optimal material for SL surgery. The study also includes different adjuvant graft mixtures and demonstrates that there is no significant difference between them and the graft itself. These results are consistent with ours and support our hypotheses, demonstrating that all types of grafts are suitable for SL. The systematic study by Papageorgiou et al. [57] included 12 articles, grouped grafts into four categories—auto-, allo-, xeno- and synthetic grafting materials—and compared them with respect to their gain of new bone. Each comparison demonstrated non-significant differences between graft types, further refuting the gold standard hypothesis for autografts and supporting the notion that all graft types can be used. These results are also consistent with those of our study.

The systematic study of Danesh-Sadi et al. (including 136 studies from before 2015) [53] also reported no significant difference between the bone gain of different types of grafts. It nevertheless proved that the autogenous graft achieves the highest new bone formation rates. In our study, AB is ranked third highest for the rate of new bone, but the two biomaterials that are superior to it (Bioglass and AlB + AB) each only have results reported by a single study and observation—Galindo-Moreno P. et al. in 2018 [33] and Pereira R. et al. in 2017 [34]. We can therefore also consider AB to be one of the most effective grafting materials in terms of the bone gain parameter. By comparing the quantity of residual graft, it also demonstrates that AB is the type of graft with the fewest residual graft particles, a finding associated with our study. This supports the conclusion that the metabolism of AB and its osteogenesis is the most rapid and complete of all the grafts.

The systematic study of Lemos analyzed the difference between grafts alone versus adjuvants. After evaluating 17 studies and comparisons regarding this subject, no significant difference was found in terms of bone gain, the quantity of connecting tissue formation, residual graft quantity, or bone resorption [58]. This would support our hypothesis, demonstrating that the addition of adjuvants with grafting biomaterials does not provide enough benefits. Likewise, the systematic review of Ortega-Mejia et al. attempts to demonstrate the advantages of adjuvants in SL. They synthesized 11 studies on this subject and, after comparing the results of each of them, also found a non-significant difference between grafts alone and with adjuvants [59]. These results also reinforce our hypothesis. On the other hand, the study by Aludden et al. attempted to demonstrate the superiority of the mixture of several types of graft compared to a single-material graft [60]. This study specifically compared IBB and IBB + AB and documented six articles dating up to 1990 on this subject. After performing the comparison, they obtained a significant difference in favor of IBB + AB compared to IBB alone. Our study has proven that IBB + AB produces a higher rate of new bone than IBB alone, but the difference is not significant. The discrepancy between Aludden et al.’s result and our systematic review may be due to the differences in study selection criteria, the fact that Aludden et al. reviewed fewer studies than us, and also that the selected studies were not from the same period.

The most important limitation of our study and the selected studies is the short follow-up period, only monitoring the results after six months of healing. These post-grafting results are therefore considered short-term. Only a few studies assess long-term results or compare short- and long-term results, such as those by Bouwman W. et al. [61], Maddalone M. et al. [62], and Shin S. et al. [63]. This type of study should be carried out in the future to validate our results over extended timeframes. Also, our study included only autogenous bone grafting materials, excluding viable, biological, and other attractive options, such as demineralized dentin matrix (DDM; also well known as an autogenous grafting material harvested after teeth removal) from the present systematic review [64]. Future systematic reviews should consider including DDM to provide a more comprehensive comparison. The third limitation is that some outcome measures rely on a single-study observation, largely due to limiting the reviewed research to a 10-year period to avoid obtaining results that may be obsolete; this limits the number of studies that may be eligible for our thesis. Finally, studies on more specific subjects, such as the difference in results between a direct and indirect sinus lift, are lacking. This prevents comparisons at this level or correlating certain results with the surgical technique used. As SL surgery becomes increasingly common, future research could explore the influence of different surgical techniques, grafting materials, and adjuvant therapies to optimize clinical outcomes.

## 5. Conclusions

This systematic review confirms that all evaluated grafting materials are viable for sinus lift surgery; however, no single graft type demonstrated clear superiority over the others. Our conclusion is supported by comparable findings regarding the rate of new bone gain, the amount of connecting tissue and residual graft, and the rate of bone resorption. The gold standard hypothesis of autograft cannot be fully supported with respect to bone gain, despite superior results to other biomaterials because the variability of connecting tissue and bone resorption is unpredictable. This study nevertheless proved that autograft’s metabolism was the fastest of all types of bone grafting materials. The hypothesis of the advantages of platelet and marrow adjuvants in sinus lift surgery could also not be validated. The observed difference in results is too small to be significant, suggesting that adjuvant substances do not provide a significant clinical advantage. Future studies should try to verify this hypothesis over the short- and long-term—over a timeframe of more than six months—in order to update current guidelines and improve the outcomes of surgical procedures.

## Figures and Tables

**Table 1:** SL New bone regeneration ratio after six months of healing time.

Author’s Information		Sample	Intervention		Histomorphological Results		Healing Time
First Author	Publication Year	Number of SL	Biomaterials	Subgroups	New Bone (%)		Months
					Average	Standard Deviation (SD)	
Pereira R. [26]	2017	12	Beta tricalcium phosphate	β-TCP	46.3	11.6	6
		9	Beta tricalcium phosphate + Autologous (1:1)	β-TCP + AB	35.0	15.8	6
		12	Autologous bone	AB	46.1	16.3	6
Traini T. [27]	2015	32	Inorganic bovine bone	IBB	34.5	0.9	6
		7	Inorganic bovine bone + Autologous (1:1)	IBB + AB	38.7	3.2	6
		22	Hydroxyapatite	HA	34.2	2.4	6
		16	Autologous bone	AB	40.1	3.2	6
Taschier S. [28]	2015	6	Inorganic bovine bone	IBB	22.7	11.3	6
		6	Inorganic bovine bone + PRF	IBB + PRF	30.7	12.4	6
Taschieri S. [29]	2016	10	Biphasic calcium phosphate (40HA-60β-TCP) + PRP	BCP + PRF	18.6	3.3	6
		10	Inorganic bovine bone + PRF	IBB + PRF	21.9	4.9	6
dos Santos Pereira R. [30]	2017	9	Β-tricalcium phosphate + Autologous (1:1)	β-TCP + AB	25.4	6.4	6
		12	Autologous	AB	38.6	10.5	6
Velasco-Ortega E. [31]	2021	8	Biphasic calcium phosphate (40HA-60β-TCP)	BCP	23.8	3.3	6
		8	Inorganic bovine bone	IBB	25.9	2.7	6
Nizam N. [32]	2018	13	Inorganic bovine bone + PRF	IBB + PRF	21.4	8.8	6
		13	Inorganic bovine bone	IBB	21.2	5.6	6
Galindo-Moreno P. [33]	2018	7	Inorganic bovine bone + Autologous (1:1)	IBB + AB	34.5	13.1	6
		7	Allogenous + Autologous (1:1)	AlB + AB	41.0	12.9	6
Pereira R. [34]	2017	10	Bioactive glass ceramic	Bioglass	42.0	7.3	6
		10	Bioactive glass ceramic + Autologous	Bioglass + AB	33.2	13.3	6
		10	Autologous	AB	35.3	14.7	6
Annibali S. [35]	2015	1	Biphasic calcium phosphate (30HA-70β-TCP)	BCP	30.2	NR	6
		1	Inorganic bovine bone	IBB	20.1	NR	6
		1	Allogenous bone	AlB	16.4	NR	6
		1	Inorganic equine bone	EB	21.9	NR	6
Sehn F. [36]	2015	17	Allogenous bone	AlB	12.5	2.5	6
		17	Allogenous + Inorganic bovine bone (2:1)	AlB + IBB	24.4	7.2	6
La Monaca G. [37]	2018	1	Allogenous bone	AlB	26.4	NR	6
		1	Inorganic bovine bone	IBB	16.1	NR	6
		1	Inorganic equine bone	EB	22.8	NR	6
		1	Biphasic calcium phosphate (30HA-70β-TCP)	BCP	20.3	NR	6
		1	Bioapatite collagen	BC	21.4	NR	6
Stacchi F. [38]	2017	26	Hydroxyapatite	HA	34.9	15	6
		26	Inorganic bovine bone	IBB	38.5	17	6
de Oliveira P. [39]	2016	7	Inorganic bovine bone	IBB	27.3	5.5	6
		7	Inorganic bovine bone + Bone marrow concentrate	IBB + BMC	38.4	12.3	6
Pasquali L. [40]	2015	8	Inorganic bovine bone	IBB	27.3	5.5	6
		8	Inorganic bovine bone + Bone marrow concentrate	IBB + BMC	55.1	20.9	6
Wildburger S. [41]	2014	7	Inorganic bovine bone	IBB	13.9	8.5	6
		6	Inorganic bovine bone + Bone marrow concentrate	IBB + BMC	13.5	5.4	6
Batas C. [42]	2019	6	Inorganic bovine bone	IBB	37.8	3.1	6
		6	Inorganic bovine bone + PRF	IBB + PRF	35.6	8.3	6
Oh J. [43]	2019	25	Inorganic bovine bone	IBB	25.1	9.6	6
		27	Biphasic calcium phosphate (60HA-40β-TCP)	BCP	28.8	7.9	6
Kivovics K. [44]	2018	11	Inorganic bovine bone	IBB	50.2	10.8	6
		12	Allogenous bone	AlB	36.3	8.0	6
Menezes D. [45]	2018	12	Bioactive glass ceramic + Autologous bone	Bioglass + AB	45.8	13.8	6
		9	Autologous bone	AB	42.0	16.6	6
Payer M. [46]	2014	5	Inorganic bovine bone	IBB	10.4	5.3	6
		6	Inorganic bovine bone + bone marrow concentrate	IBB + BMC	14.2	3.6	6
Jelusic A. [47]	2017	30	Biphasic calcium phosphate (60HA-40β-TCP)	BCP	38.4	19.4	6
		30	Beta tricalcium phosphate	β-TCP	36.2	10.4	6
Meimandi G. [48]	2017	10	Hydroxyapatite + PRF	HA + PRF	30.3	8.5	6
		10	Hydroxyapatite	HA	25.3	7.3	6
Kilic C. [49]	2017	9	Beta tricalcium phosphate	β-TCP	32	6.3	6
		8	Beta tricalcium phosphate + Autologous bone	β-TCP + AB	33.4	10.4	6

**Table 2:** Post-SL Connective tissue and residual graft.

Author’s Information		Sample	Intervention		Histomorphological Results				Healing Time
First Author	Publication Year	SL Number	Biomaterials	Subgroups	Connective Tissue Quantity (%)	Standard Deviation (SD)	Residual Graft Quantity (%)	Standard Deviation (SD)	Months
Mordenfeld A. [50]	2014	14	Inorganic bovine bone	IBB	42.0	5.5	NR	NR	7.5
		14	Inorganic bovine bone + Autogenous	IBB + AB	46.0	6.5	NR	NR	7.5
La Monaca G. [47]	2018	1	Allogenic	AlB	47.8	NR	20.1	NR	6
		1	Inorganic bovine bone	IBB	46.7	NR	37.2	NR	6
		1	Inorganic equine bone	EB	47.1	NR	30.1	NR	6
		1	Biphasic calcium phosphate (30HA/70β-TCP)	BCP	41.8	NR	37.9	NR	6
		1	Hydroxyapatite	HA	53.3	NR	25.3	NR	6
Kilic C. [49]	2017	9	Beta tricalcium phosphate	Β-TCP	36.2	10.6	30.4	10.3	6
		9	Beta tricalcium phosphate + PRF	β-TCP + PRF	35.3	10.8	32.7	7.5	6
Correia F. [51]	2021	6	Autogenous	AB	42.7	2.9	NR	NR	6
		6	Inorganic porcine bone	IPB	44.0	2.9	NR	NR	6
Traini T. [25]	2015	32	Inorganic bovine bone	IBB	36.4	2.3	32.8	2.1	6
		22	Hydroxyapatite	HA	35.6	2.3	37.1	3.8	6
		16	Autogenous	AB	40.0	2.1	18.3	2.3	6
		7	Inorganic bovine bone + Autogenous (1:1)	IBB + AB	45.6	5.0	14.4	2.1	6
		16	Bioactive glass ceramic	Bioglass	49.0	1.8	20.0	2.4	6
Starch-Jensen T. [52]	2018	10	Autogenous	AB	58.4	NR	10.8	NR	6
		10	Biphasic calcium phosphate (30HA/70β-TCP)	BCP	38.9	NR	32.9	NR	6
Danesh Sadi S. [53]	2017	NR	Autogenous	AB	47.6	NR	17.7	NR	4.5–9
		NR	Allogenic	AlB	50.0	NR	25.3	NR	4.5–9
		NR	Inorganic bovine bone	ABB	44.9	NR	29.3	NR	4.5–9
		NR	Inorganic bovine bone + Autogenous (1:1)	IBB + AB	47.4	NR	22.8	NR	4.5–9
		NR	Allogenic + Autogenous (1:1)	AlB + AB	48.3	NR	23.5	NR	4.5–9
		NR	Inorganic bovine bone + Allogenic (1:1)	IBB + AlB	57.8	NR	25.3	NR	4.5–9
Annibali S. [35]	2015	1	Biphasic calcium phosphate (30HA/70β-TCP)	BCP	40.7	NR	29.1	NR	6
		1	Inorganic bovine bone	ABB	60.8	NR	19.1	NR	6
		1	Inorganic equine bone	EB	54.9	NR	23.2	NR	6
		1	Allogenic	AlB	55.1	NR	18.5	NR	6
Galindo-Moreno P. [33]	2018	7	Inorganic bovine bone + Autogenous (1:1)	IBB + AB	43.8	19.9	21.7	17.9	6
		7	Allogenic + Autogenous (1:1)	AlB + AB	49.0	14.3	9.3	7.7	6
Nizam N. [32]	2018	13	Inorganic bovine bone	IBB	45.9	8.4	32.8	5.9	6
		13	Inorganic bovine bone + PRF	IBB + PRF	52.7	12.5	25.9	9.5	6

**Table 3:** Bone resorption ratio after six-month healing period.

Author’s Information		Sample	Intervention		Radiological Results		Healing Time
First author	Publication Year	SL Number	Biomaterials	Subgroups	Bone Resorption Ratio (%)	Standard Deviation (SD)	Months
Starch-Jensen T. [52]	2018	14	Hydroxyapatite	HA	28.5	NR	7
		14	Inorganic bovine bone	IBB	22.4	NR	7
Mordenfeld A. [50]	2014	14	Inorganic bovine bone + Autogenous	IBB + AB	37	19.9	7.5
		14	Inorganic bovine bone	IBB	46.9	23.5	7.5
Pereira R. [54]	2018	12	Bioactive glass ceramic	Bioglass	44	16	6
		9	Bioactive glass ceramic + Autogenous (1:1)	Bioglass + AB	37.9	18.9	6
		12	Autogenous	AB	45.7	18,5	6
Pereira R. [30]	2017	12	Beta tricalcium phosphate	β-TCP	43.8	NR	6
		12	Autogenous	AB	45.7	NR	6
		9	Beta tricalcium phosphate + Autogenous (1:1)	β-TCP + AB	38.3	NR	6
Sehn F. [36]	2015	17	Allogenic	AlB	28.3	11.3	6
Xavier S. [55]	2016	15	Allogenic	AlB	31.2	19.9	6
		15	Inorganic bovine bone	IBB	12.2	2.3	6

## Data Availability

The original contributions presented in this study are included in the article/Supplementary Material. Further inquiries can be directed to the corresponding authors.

## Supplementary Materials

The following supporting information can be downloaded at: https://www.mdpi.com/article/10.3390/jfb16040133/s1.
