# Supplementary material for: Insights into Sinus-Lift Bone Grafting Materials: What’s Changed?
Source: J Funct Biomater. 2025 Apr 7;16(4):133. doi: 10.3390/jfb16040133 (PMC12028325; doi:10.3390/jfb16040133)
Supplement: Supplementary file 1 [file jfb-16-00133-s001.zip › jfb-3374484-supplementary.pdf]

Supplementary S1: ANOVA Fischer test new bone regeneration ratio comparison

Type de greffe / Fisher (LSD) / Analyse des différences entre les modalités avec un intervalle de confiance à 95% (Moyenne) :

Contraste	Différence	Différence standardisée	Valeur critique	Pr > Diff	Significatif
Bioglass vs BCP + PRF	23,400	2,287	2,023	**0,028**	Oui
Bioglass vs BC	20,600	2,013	2,023	0,051	Non
Bioglass vs EB	19,650	1,920	2,023	0,062	Non
Bioglass vs AlB	19,100	1,867	2,023	0,069	Non
Bioglass vs AlB + ABB	17,600	1,720	2,023	0,093	Non
Bioglass vs ABB	15,500	1,515	2,023	0,138	Non
Bioglass vs ABB + PRF	14,600	1,427	2,023	0,162	Non
Bioglass vs BCP	13,700	1,339	2,023	0,188	Non
Bioglass vs ABB + BMC	11,700	1,143	2,023	0,260	Non
Bioglass vs HA + PRF	11,700	1,143	2,023	0,260	Non
Bioglass vs β-TCP + AB	10,733	1,049	2,023	0,301	Non
Bioglass vs HA	10,533	1,029	2,023	0,310	Non
Bioglass vs ABB + AB	5,400	0,528	2,023	0,601	Non
Bioglass vs β-TCP	3,833	0,375	2,023	0,710	Non
Bioglass vs Bioglass + /	2,500	0,244	2,023	0,808	Non
Bioglass vs AB	1,580	0,154	2,023	0,878	Non
Bioglass vs AlB + AB	1,000	0,098	2,023	0,923	Non
AlB + AB vs BCP + PRF	22,400	2,189	2,023	**0,035**	Oui
AlB + AB vs BC	19,600	1,915	2,023	0,063	Non
AlB + AB vs EB	18,650	1,823	2,023	0,076	Non
AlB + AB vs AlB	18,100	1,769	2,023	0,085	Non
AlB + AB vs AlB + ABB	16,600	1,622	2,023	0,113	Non
AlB + AB vs ABB	14,500	1,417	2,023	0,164	Non
AlB + AB vs ABB + PRF	13,600	1,329	2,023	0,192	Non
AlB + AB vs BCP	12,700	1,241	2,023	0,222	Non
AlB + AB vs ABB + BMC	10,700	1,046	2,023	0,302	Non
AlB + AB vs HA + PRF	10,700	1,046	2,023	0,302	Non
AlB + AB vs β-TCP + AB	9,733	0,951	2,023	0,347	Non
AlB + AB vs HA	9,533	0,932	2,023	0,357	Non
AlB + AB vs ABB + AB	4,400	0,430	2,023	0,670	Non
AlB + AB vs β-TCP	2,833	0,277	2,023	0,783	Non
AlB + AB vs Bioglass + /	1,500	0,147	2,023	0,884	Non
AlB + AB vs AB	0,580	0,057	2,023	0,955	Non
AB vs BCP + PRF	21,820	2,132	2,023	**0,039**	Oui
AB vs BC	19,020	1,859	2,023	0,071	Non
AB vs EB	18,070	1,766	2,023	0,085	Non
AB vs AlB	17,520	1,712	2,023	0,095	Non
AB vs AlB + ABB	16,020	1,566	2,023	0,126	Non
AB vs ABB	13,920	1,360	2,023	0,182	Non
AB vs ABB + PRF	13,020	1,272	2,023	0,211	Non
AB vs BCP	12,120	1,184	2,023	0,243	Non
AB vs ABB + BMC	10,120	0,989	2,023	0,329	Non
AB vs HA + PRF	10,120	0,989	2,023	0,329	Non
AB vs β-TCP + AB	9,153	0,895	2,023	0,377	Non
AB vs HA	8,953	0,875	2,023	0,387	Non
AB vs ABB + AB	3,820	0,373	2,023	0,711	Non
AB vs β-TCP	2,253	0,220	2,023	0,827	Non
AB vs Bioglass + AB	0,920	0,090	2,023	0,929	Non
Bioglass + AB vs BCP +	20,900	2,042	2,023	**0,048**	Oui
Bioglass + AB vs BC	18,100	1,769	2,023	0,085	Non
Bioglass + AB vs EB	17,150	1,676	2,023	0,102	Non
Bioglass + AB vs AlB	16,600	1,622	2,023	0,113	Non
Bioglass + AB vs AlB + /	15,100	1,476	2,023	0,148	Non
Bioglass + AB vs ABB	13,000	1,270	2,023	0,211	Non
Bioglass + AB vs ABB +	12,100	1,182	2,023	0,244	Non
Bioglass + AB vs BCP	11,200	1,095	2,023	0,280	Non
Bioglass + AB vs ABB +	9,200	0,899	2,023	0,374	Non
Bioglass + AB vs HA + F	9,200	0,899	2,023	0,374	Non
Bioglass + AB vs β-TCP	8,233	0,805	2,023	0,426	Non
Bioglass + AB vs HA	8,033	0,785	2,023	0,437	Non
Bioglass + AB vs ABB +	2,900	0,283	2,023	0,778	Non
Bioglass + AB vs β-TCP	1,333	0,130	2,023	0,897	Non
β-TCP vs BCP + PRF	19,567	1,912	2,023	0,063	Non
β-TCP vs BC	16,767	1,639	2,023	0,109	Non
β-TCP vs EB	15,817	1,546	2,023	0,130	Non
β-TCP vs AlB	15,267	1,492	2,023	0,144	Non
β-TCP vs AlB + ABB	13,767	1,345	2,023	0,186	Non
β-TCP vs ABB	11,667	1,140	2,023	0,261	Non
β-TCP vs ABB + PRF	10,767	1,052	2,023	0,299	Non
β-TCP vs BCP	9,867	0,964	2,023	0,341	Non
β-TCP vs ABB + BMC	7,867	0,769	2,023	0,447	Non
β-TCP vs HA + PRF	7,867	0,769	2,023	0,447	Non
β-TCP vs β-TCP + AB	6,900	0,674	2,023	0,504	Non
β-TCP vs HA	6,700	0,655	2,023	0,516	Non
β-TCP vs ABB + AB	1,567	0,153	2,023	0,879	Non

ABB + AB vs BCP + PRF	18,000	1,759	2,023	0,086	Non
ABB + AB vs BC	15,200	1,485	2,023	0,145	Non
ABB + AB vs EB	14,250	1,393	2,023	0,172	Non
ABB + AB vs AlB	13,700	1,339	2,023	0,188	Non
ABB + AB vs AlB + ABB	12,200	1,192	2,023	0,240	Non
ABB + AB vs ABB	10,100	0,987	2,023	0,330	Non
ABB + AB vs ABB + PRF	9,200	0,899	2,023	0,374	Non
ABB + AB vs BCP	8,300	0,811	2,023	0,422	Non
ABB + AB vs ABB + BMC	6,300	0,616	2,023	0,542	Non
ABB + AB vs HA + PRF	6,300	0,616	2,023	0,542	Non
ABB + AB vs β-TCP + AI	5,333	0,521	2,023	0,605	Non
ABB + AB vs HA	5,133	0,502	2,023	0,619	Non
HA vs BCP + PRF	12,867	1,257	2,023	0,216	Non
HA vs BC	10,067	0,984	2,023	0,331	Non
HA vs EB	9,117	0,891	2,023	0,378	Non
HA vs AlB	8,567	0,837	2,023	0,408	Non
HA vs AlB + ABB	7,067	0,691	2,023	0,494	Non
HA vs ABB	4,967	0,485	2,023	0,630	Non
HA vs ABB + PRF	4,067	0,397	2,023	0,693	Non
HA vs BCP	3,167	0,309	2,023	0,759	Non
HA vs ABB + BMC	1,167	0,114	2,023	0,910	Non
HA vs HA + PRF	1,167	0,114	2,023	0,910	Non
HA vs β-TCP + AB	0,200	0,020	2,023	0,985	Non
β-TCP + AB vs BCP + PI	12,667	1,238	2,023	0,223	Non
β-TCP + AB vs BC	9,867	0,964	2,023	0,341	Non
β-TCP + AB vs EB	8,917	0,871	2,023	0,389	Non
β-TCP + AB vs AlB	8,367	0,818	2,023	0,419	Non
β-TCP + AB vs AlB + AB	6,867	0,671	2,023	0,506	Non
β-TCP + AB vs ABB	4,767	0,466	2,023	0,644	Non
β-TCP + AB vs ABB + PI	3,867	0,378	2,023	0,708	Non
β-TCP + AB vs BCP	2,967	0,290	2,023	0,773	Non
β-TCP + AB vs ABB + BI	0,967	0,094	2,023	0,925	Non
β-TCP + AB vs HA + PRI	0,967	0,094	2,023	0,925	Non
HA + PRF vs BCP + PRF	11,700	1,143	2,023	0,260	Non
HA + PRF vs BC	8,900	0,870	2,023	0,390	Non
HA + PRF vs EB	7,950	0,777	2,023	0,442	Non
HA + PRF vs AlB	7,400	0,723	2,023	0,474	Non
HA + PRF vs AlB + ABB	5,900	0,577	2,023	0,568	Non
HA + PRF vs ABB	3,800	0,371	2,023	0,712	Non
HA + PRF vs ABB + PRF	2,900	0,283	2,023	0,778	Non
HA + PRF vs BCP	2,000	0,195	2,023	0,846	Non
HA + PRF vs ABB + BMC	0,000	0,000	2,023	1,000	Non
ABB + BMC vs BCP + PI	11,700	1,143	2,023	0,260	Non
ABB + BMC vs BC	8,900	0,870	2,023	0,390	Non
ABB + BMC vs EB	7,950	0,777	2,023	0,442	Non
ABB + BMC vs AlB	7,400	0,723	2,023	0,474	Non
ABB + BMC vs AlB + AB	5,900	0,577	2,023	0,568	Non
ABB + BMC vs ABB	3,800	0,371	2,023	0,712	Non
ABB + BMC vs ABB + PI	2,900	0,283	2,023	0,778	Non
ABB + BMC vs BCP	2,000	0,195	2,023	0,846	Non
BCP vs BCP + PRF	9,700	0,948	2,023	0,349	Non
BCP vs BC	6,900	0,674	2,023	0,504	Non
BCP vs EB	5,950	0,581	2,023	0,564	Non
BCP vs AlB	5,400	0,528	2,023	0,601	Non
BCP vs AlB + ABB	3,900	0,381	2,023	0,705	Non
BCP vs ABB	1,800	0,176	2,023	0,861	Non
BCP vs ABB + PRF	0,900	0,088	2,023	0,930	Non
ABB + PRF vs BCP + PR	8,800	0,860	2,023	0,395	Non
ABB + PRF vs BC	6,000	0,586	2,023	0,561	Non
ABB + PRF vs EB	5,050	0,494	2,023	0,624	Non
ABB + PRF vs AlB	4,500	0,440	2,023	0,663	Non
ABB + PRF vs AlB + ABE	3,000	0,293	2,023	0,771	Non
ABB + PRF vs ABB	0,900	0,088	2,023	0,930	Non
ABB vs BCP + PRF	7,900	0,772	2,023	0,445	Non
ABB vs BC	5,100	0,498	2,023	0,621	Non
ABB vs EB	4,150	0,406	2,023	0,687	Non
ABB vs AlB	3,600	0,352	2,023	0,727	Non
ABB vs AlB + ABB	2,100	0,205	2,023	0,838	Non
AlB + ABB vs BCP + PRI	5,800	0,567	2,023	0,574	Non
AlB + ABB vs BC	3,000	0,293	2,023	0,771	Non
AlB + ABB vs EB	2,050	0,200	2,023	0,842	Non
AlB + ABB vs AlB	1,500	0,147	2,023	0,884	Non
AlB vs BCP + PRF	4,300	0,420	2,023	0,677	Non
AlB vs BC	1,500	0,147	2,023	0,884	Non
AlB vs EB	0,550	0,054	2,023	0,957	Non
EB vs BCP + PRF	3,750	0,366	2,023	0,716	Non
EB vs BC	0,950	0,093	2,023	0,927	Non
BC vs BCP + PRF	2,800	0,274	2,023	0,786	Non
Différence significative minimale :			7,527		

Supplementary S2: ANOVA Fischer test connective tissue quantity and residual graft ratio comparison

Type de greffe / Fisher (LSD) / Analyse des différences entre les modalités avec un intervalle de confiance à 95% (Quantité de tissus de connexion (%)) :

Contraste	Différence	Différence standardisée	Valeur critique	Pr > Diff	Significatif
ABB + AlB vs β-TCP + PRF	22,500	2,399	2,110	**0,028**	Oui
ABB + AlB vs β-TCP	21,600	2,303	2,110	**0,034**	Oui
ABB + AlB vs BCP	17,333	2,263	2,110	**0,037**	Oui
ABB + AlB vs ABB + AB	14,000	1,492	2,110	0,154	Non
ABB + AlB vs APB	13,800	1,471	2,110	0,160	Non
ABB + AlB vs HA	13,350	1,643	2,110	0,119	Non
ABB + AlB vs ABB	11,683	1,631	2,110	0,121	Non
ABB + AlB vs ABB + AB	11,467	1,497	2,110	0,153	Non
ABB + AlB vs AB	10,625	1,433	2,110	0,170	Non
ABB + AlB vs AlB + AB	9,150	1,126	2,110	0,276	Non
ABB + AlB vs Bioglass	8,800	0,938	2,110	0,361	Non
ABB + AlB vs AlB	6,833	0,892	2,110	0,385	Non
ABB + AlB vs EB	6,800	0,837	2,110	0,414	Non
ABB + AlB vs ABB + PRF	5,100	0,544	2,110	0,594	Non
ABB + PRF vs β-TCP + PRF	17,400	1,855	2,110	0,081	Non
ABB + PRF vs β-TCP	16,500	1,759	2,110	0,097	Non
ABB + PRF vs BCP	12,233	1,597	2,110	0,129	Non
ABB + PRF vs ABB + AB	8,900	0,949	2,110	0,356	Non
ABB + PRF vs APB	8,700	0,927	2,110	0,367	Non
ABB + PRF vs HA	8,250	1,016	2,110	0,324	Non
ABB + PRF vs ABB	6,583	0,919	2,110	0,371	Non
ABB + PRF vs ABB + AB	6,367	0,831	2,110	0,417	Non
ABB + PRF vs AB	5,525	0,745	2,110	0,466	Non
ABB + PRF vs AlB + AB	4,050	0,499	2,110	0,624	Non
ABB + PRF vs Bioglass	3,700	0,394	2,110	0,698	Non
ABB + PRF vs AlB	1,733	0,226	2,110	0,824	Non
ABB + PRF vs EB	1,700	0,209	2,110	0,837	Non
EB vs β-TCP + PRF	15,700	1,933	2,110	0,070	Non
EB vs β-TCP	14,800	1,822	2,110	0,086	Non
EB vs BCP	10,533	1,740	2,110	0,100	Non
EB vs ABB + AB	7,200	0,886	2,110	0,388	Non
EB vs APB	7,000	0,862	2,110	0,401	Non
EB vs HA	6,550	0,987	2,110	0,337	Non
EB vs ABB	4,883	0,902	2,110	0,380	Non
EB vs ABB + AB	4,667	0,771	2,110	0,451	Non
EB vs AB	3,825	0,666	2,110	0,514	Non
EB vs AlB + AB	2,350	0,354	2,110	0,727	Non
EB vs Bioglass	2,000	0,246	2,110	0,808	Non
EB vs AlB	0,033	0,006	2,110	0,996	Non
AlB vs β-TCP + PRF	15,667	2,045	2,110	0,057	Non
AlB vs β-TCP	14,767	1,928	2,110	0,071	Non
AlB vs BCP	10,500	1,939	2,110	0,069	Non
AlB vs ABB + AB	7,167	0,936	2,110	0,363	Non
AlB vs APB	6,967	0,910	2,110	0,376	Non
AlB vs HA	6,517	1,076	2,110	0,297	Non
AlB vs ABB	4,850	1,034	2,110	0,316	Non
AlB vs ABB + AB	4,633	0,856	2,110	0,404	Non
AlB vs AB	3,792	0,748	2,110	0,464	Non
AlB vs AlB + AB	2,317	0,383	2,110	0,707	Non
AlB vs Bioglass	1,967	0,257	2,110	0,800	Non
Bioglass vs β-TCP + PRF	13,700	1,460	2,110	0,162	Non
Bioglass vs β-TCP	12,800	1,365	2,110	0,190	Non
Bioglass vs BCP	8,533	1,114	2,110	0,281	Non
Bioglass vs ABB + AB	5,200	0,554	2,110	0,587	Non
Bioglass vs APB	5,000	0,533	2,110	0,601	Non
Bioglass vs HA	4,550	0,560	2,110	0,583	Non
Bioglass vs ABB	2,883	0,402	2,110	0,692	Non
Bioglass vs ABB + AB	2,667	0,348	2,110	0,732	Non
Bioglass vs AB	1,825	0,246	2,110	0,809	Non
Bioglass vs AlB + AB	0,350	0,043	2,110	0,966	Non
AlB + AB vs β-TCP + PRF	13,350	1,643	2,110	0,119	Non
AlB + AB vs β-TCP	12,450	1,533	2,110	0,144	Non
AlB + AB vs BCP	8,183	1,351	2,110	0,194	Non
AlB + AB vs ABB + AB	4,850	0,597	2,110	0,558	Non
AlB + AB vs APB	4,650	0,572	2,110	0,575	Non
AlB + AB vs HA	4,200	0,633	2,110	0,535	Non
AlB + AB vs ABB	2,533	0,468	2,110	0,646	Non
AlB + AB vs ABB + AB	2,317	0,383	2,110	0,707	Non
AlB + AB vs AB	1,475	0,257	2,110	0,800	Non
AB vs β-TCP + PRF	11,875	1,601	2,110	0,128	Non
AB vs β-TCP	10,975	1,480	2,110	0,157	Non
AB vs BCP	6,708	1,324	2,110	0,203	Non
AB vs ABB + AB	3,375	0,455	2,110	0,655	Non
AB vs APB	3,175	0,428	2,110	0,674	Non
AB vs HA	2,725	0,474	2,110	0,641	Non
AB vs ABB	1,058	0,247	2,110	0,808	Non
AB vs ABB + AB	0,842	0,166	2,110	0,870	Non
ABB + AB vs β-TCP + PRF	11,033	1,441	2,110	0,168	Non
ABB + AB vs β-TCP	10,133	1,323	2,110	0,203	Non
ABB + AB vs BCP	5,867	1,083	2,110	0,294	Non
ABB + AB vs ABB + AB	2,533	0,331	2,110	0,745	Non
ABB + AB vs APB	2,333	0,305	2,110	0,764	Non
ABB + AB vs HA	1,883	0,311	2,110	0,760	Non
ABB + AB vs ABB	0,217	0,046	2,110	0,964	Non
ABB vs β-TCP + PRF	10,817	1,510	2,110	0,149	Non
ABB vs β-TCP	9,917	1,384	2,110	0,184	Non
ABB vs BCP	5,650	1,205	2,110	0,245	Non
ABB vs ABB + AB	2,317	0,323	2,110	0,750	Non
ABB vs APB	2,117	0,295	2,110	0,771	Non
ABB vs HA	1,667	0,308	2,110	0,762	Non
HA vs β-TCP + PRF	9,150	1,126	2,110	0,276	Non
HA vs β-TCP	8,250	1,016	2,110	0,324	Non
HA vs BCP	3,983	0,658	2,110	0,519	Non
HA vs ABB + AB	0,650	0,080	2,110	0,937	Non
HA vs APB	0,450	0,055	2,110	0,956	Non
APB vs β-TCP + PRF	8,700	0,927	2,110	0,367	Non
APB vs β-TCP	7,800	0,832	2,110	0,417	Non
APB vs BCP	3,533	0,461	2,110	0,650	Non
APB vs ABB + AB	0,200	0,021	2,110	0,983	Non
ABB + AB vs β-TCP + PRF	8,500	0,906	2,110	0,378	Non
ABB + AB vs β-TCP	7,600	0,810	2,110	0,429	Non
ABB + AB vs BCP	3,333	0,435	2,110	0,669	Non
BCP vs β-TCP + PRF	5,167	0,675	2,110	0,509	Non
BCP vs β-TCP	4,267	0,557	2,110	0,585	Non
β-TCP vs β-TCP + PRF	0,900	0,096	2,110	0,925	Non
Différence significative minimale :			8,08		

Supplementary S3: ANOVA Fischer test bone residual graft ratio comparison

**Type de greffe / Fisher (LSD) / Analyse des différences entre les modalités avec un intervalle de confiance à 95% (Quantié greffe rési**					
	Différence	Différence standardisée	Valeur critique	Pr > Diff	Significatif
BCP vs AB	17,700	2,546	2,145	**0,023**	Oui
BCP vs AlB + AB	16,900	2,430	2,145	**0,029**	Oui
BCP vs ABB + AB	14,700	2,114	2,145	0,053	Non
BCP vs Bioglass	13,300	1,913	2,145	0,076	Non
BCP vs AlB	12,000	1,726	2,145	0,106	Non
BCP vs ABB + AB	11,600	1,668	2,145	0,117	Non
BCP vs ABB + AlB	8,000	1,151	2,145	0,269	Non
BCP vs ABB + PRF	7,400	1,064	2,145	0,305	Non
BCP vs EB	6,650	0,956	2,145	0,355	Non
BCP vs ABB	3,060	0,440	2,145	0,667	Non
BCP vs β-TCP	2,900	0,417	2,145	0,683	Non
BCP vs HA	2,100	0,302	2,145	0,767	Non
BCP vs β-TCP + PRF	0,600	0,086	2,145	0,932	Non
β-TCP + PRF vs AB	17,100	2,459	2,145	**0,028**	Oui
β-TCP + PRF vs AlB + AB	16,300	2,344	2,145	**0,034**	Oui
β-TCP + PRF vs ABB + AB	14,100	2,028	2,145	0,062	Non
β-TCP + PRF vs Bioglass	12,700	1,826	2,145	0,089	Non
β-TCP + PRF vs AlB	11,400	1,639	2,145	0,123	Non
β-TCP + PRF vs ABB + AB	11,000	1,582	2,145	0,136	Non
β-TCP + PRF vs ABB + AlB	7,400	1,064	2,145	0,305	Non
β-TCP + PRF vs ABB + PRF	6,800	0,978	2,145	0,345	Non
β-TCP + PRF vs EB	6,050	0,870	2,145	0,399	Non
β-TCP + PRF vs ABB	2,460	0,354	2,145	0,729	Non
β-TCP + PRF vs β-TCP	2,300	0,331	2,145	0,746	Non
β-TCP + PRF vs HA	1,500	0,216	2,145	0,832	Non
HA vs AB	15,600	2,243	2,145	**0,042**	Oui
HA vs AlB + AB	14,800	2,128	2,145	0,052	Non
HA vs ABB + AB	12,600	1,812	2,145	0,091	Non
HA vs Bioglass	11,200	1,611	2,145	0,130	Non
HA vs AlB	9,900	1,424	2,145	0,176	Non
HA vs ABB + AB	9,500	1,366	2,145	0,193	Non
HA vs ABB + AlB	5,900	0,849	2,145	0,410	Non
HA vs ABB + PRF	5,300	0,762	2,145	0,459	Non
HA vs EB	4,550	0,654	2,145	0,523	Non
HA vs ABB	0,960	0,138	2,145	0,892	Non
HA vs β-TCP	0,800	0,115	2,145	0,910	Non
β-TCP vs AB	14,800	2,128	2,145	0,052	Non
β-TCP vs AlB + AB	14,000	2,013	2,145	0,064	Non
β-TCP vs ABB + AB	11,800	1,697	2,145	0,112	Non
β-TCP vs Bioglass	10,400	1,496	2,145	0,157	Non
β-TCP vs AlB	9,100	1,309	2,145	0,212	Non
β-TCP vs ABB + AB	8,700	1,251	2,145	0,231	Non
β-TCP vs ABB + AlB	5,100	0,733	2,145	0,475	Non
β-TCP vs ABB + PRF	4,500	0,647	2,145	0,528	Non
β-TCP vs EB	3,750	0,539	2,145	0,598	Non
β-TCP vs ABB	0,160	0,023	2,145	0,982	Non
ABB vs AB	14,640	2,105	2,145	0,054	Non
ABB vs AlB + AB	13,840	1,990	2,145	0,066	Non
ABB vs ABB + AB	11,640	1,674	2,145	0,116	Non
ABB vs Bioglass	10,240	1,473	2,145	0,163	Non
ABB vs AlB	8,940	1,286	2,145	0,219	Non
ABB vs ABB + AB	8,540	1,228	2,145	0,240	Non
ABB vs ABB + AlB	4,940	0,710	2,145	0,489	Non
ABB vs ABB + PRF	4,340	0,624	2,145	0,543	Non
ABB vs EB	3,590	0,516	2,145	0,614	Non
EB vs AB	11,050	1,589	2,145	0,134	Non
EB vs AlB + AB	10,250	1,474	2,145	0,163	Non
EB vs ABB + AB	8,050	1,158	2,145	0,266	Non
EB vs Bioglass	6,650	0,956	2,145	0,355	Non
EB vs AlB	5,350	0,769	2,145	0,454	Non
EB vs ABB + AB	4,950	0,712	2,145	0,488	Non
EB vs ABB + AlB	1,350	0,194	2,145	0,849	Non
EB vs ABB + PRF	0,750	0,108	2,145	0,916	Non
ABB + PRF vs AB	10,300	1,481	2,145	0,161	Non
ABB + PRF vs AlB + AB	9,500	1,366	2,145	0,193	Non
ABB + PRF vs ABB + AB	7,300	1,050	2,145	0,312	Non
ABB + PRF vs Bioglass	5,900	0,849	2,145	0,410	Non
ABB + PRF vs AlB	4,600	0,662	2,145	0,519	Non
ABB + PRF vs ABB + AB	4,200	0,604	2,145	0,555	Non
ABB + PRF vs ABB + AlB	0,600	0,086	2,145	0,932	Non
ABB + AlB vs AB	9,700	1,395	2,145	0,185	Non
ABB + AlB vs AlB + AB	8,900	1,280	2,145	0,221	Non
ABB + AlB vs ABB + AB	6,700	0,964	2,145	0,352	Non
ABB + AlB vs Bioglass	5,300	0,762	2,145	0,459	Non
ABB + AlB vs AlB	4,000	0,575	2,145	0,574	Non
ABB + AlB vs ABB + AB	3,600	0,518	2,145	0,613	Non
ABB + AB vs AB	6,100	0,877	2,145	0,395	Non
ABB + AB vs AlB + AB	5,300	0,762	2,145	0,459	Non
ABB + AB vs ABB + AB	3,100	0,446	2,145	0,663	Non
ABB + AB vs Bioglass	1,700	0,244	2,145	0,810	Non
ABB + AB vs AlB	0,400	0,058	2,145	0,955	Non
AlB vs AB	5,700	0,820	2,145	0,426	Non
AlB vs AlB + AB	4,900	0,705	2,145	0,493	Non
AlB vs ABB + AB	2,700	0,388	2,145	0,704	Non
AlB vs Bioglass	1,300	0,187	2,145	0,854	Non
Bioglass vs AB	4,400	0,633	2,145	0,537	Non
Bioglass vs AlB + AB	3,600	0,518	2,145	0,613	Non
Bioglass vs ABB + AB	1,400	0,201	2,145	0,843	Non
ABB + AB vs AB	3,000	0,431	2,145	0,673	Non
ABB + AB vs AlB + AB	2,200	0,316	2,145	0,756	Non
AlB + AB vs AB	0,800	0,115	2,145	0,910	Non
Différence significative minimale :			8,228		

Supplementary S4: ANOVA Fischer test bone resorption ratio graft ratio comparison

Type de greffe / Fisher (LSD) / Analyse des différences entre les modalités avec un intervalle de confiance à 95% (Moyenne Résorption osseuse (%)) :					
Contraste	Différence	Différence standardisée	Valeur critique	Pr > Diff	Significatif
AB vs ABB	18,533	1,605	2,776	0,184	Non
AB vs HA	17,200	1,110	2,776	0,329	Non
AB vs AlB	15,950	1,261	2,776	0,276	Non
AB vs ABB + AB	8,700	0,561	2,776	0,604	Non
AB vs Bioglass + AB	7,800	0,503	2,776	0,641	Non
AB vs β-TCP + AB	7,400	0,478	2,776	0,658	Non
AB vs β-TCP	1,900	0,123	2,776	0,908	Non
AB vs Bioglass	1,700	0,110	2,776	0,918	Non
Bioglass vs ABB	16,833	1,152	2,776	0,313	Non
Bioglass vs HA	15,500	0,866	2,776	0,435	Non
Bioglass vs AlB	14,250	0,920	2,776	0,410	Non
Bioglass vs ABB + AB	7,000	0,391	2,776	0,716	Non
Bioglass vs Bioglass + AB	6,100	0,341	2,776	0,750	Non
Bioglass vs β-TCP + AB	5,700	0,319	2,776	0,766	Non
Bioglass vs β-TCP	0,200	0,011	2,776	0,992	Non
β-TCP vs ABB	16,633	1,139	2,776	0,318	Non
β-TCP vs HA	15,300	0,855	2,776	0,441	Non
β-TCP vs AlB	14,050	0,907	2,776	0,416	Non
β-TCP vs ABB + AB	6,800	0,380	2,776	0,723	Non
β-TCP vs Bioglass + AB	5,900	0,330	2,776	0,758	Non
β-TCP vs β-TCP + AB	5,500	0,307	2,776	0,774	Non
β-TCP + AB vs ABB	11,133	0,762	2,776	0,488	Non
β-TCP + AB vs HA	9,800	0,548	2,776	0,613	Non
β-TCP + AB vs AlB	8,550	0,552	2,776	0,610	Non
β-TCP + AB vs ABB + AB	1,300	0,073	2,776	0,946	Non
β-TCP + AB vs Bioglass + AB	0,400	0,022	2,776	0,983	Non
Bioglass + AB vs ABB	10,733	0,735	2,776	0,503	Non
Bioglass + AB vs HA	9,400	0,525	2,776	0,627	Non
Bioglass + AB vs AlB	8,150	0,526	2,776	0,627	Non
Bioglass + AB vs ABB + AB	0,900	0,050	2,776	0,962	Non
ABB + AB vs ABB	9,833	0,673	2,776	0,538	Non
ABB + AB vs HA	8,500	0,475	2,776	0,660	Non
ABB + AB vs AlB	7,250	0,468	2,776	0,664	Non
AlB vs ABB	2,583	0,224	2,776	0,834	Non
AlB vs HA	1,250	0,081	2,776	0,940	Non
HA vs ABB	1,333	0,091	2,776	0,932	Non
Différence significative minimale :			28,682		

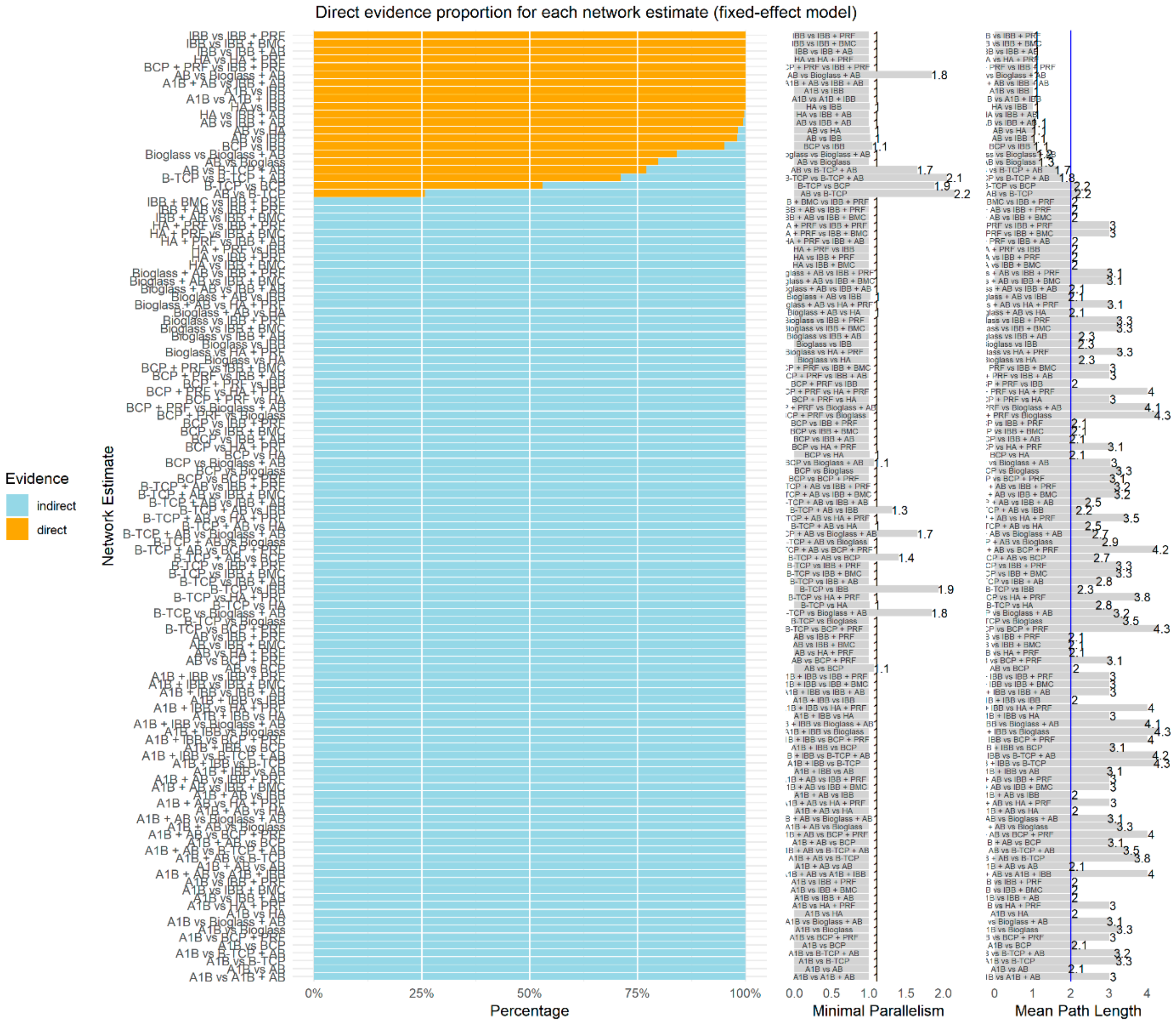

Direct evidence proportion for each network estimate (fixed-effect model)
Evidence
indirect
direct
Network Estimate
0%
25%
50%
75%
100%
Percentage
0.0
0.5
1.0
1.5
2.0
Minimal Parallelism
0
1
2
3
4
Mean Path Length

Figure S1. Direct and indirect evidence for NBRR.

Table S1. League table for NBRR.

	**V1**	**V2**	**V3**	**V4**	**V5**	**V6**	**V7**	**V8**	**V9**	**V10**	**V11**	**V12**	**V13**	**V14**	**V15**	**V16**
1	A1B	.	-11.90 (-20.18; -3.62)	.	.	.	.	.	.	.	.	.	-13.90 (-24.70; -3.10)	.	.	.
2	-24.05 (-44.36; -3.73)	A1B + AB	.	.	.	.	.	.	.	.	.	.	.	6.50 (-9.02; 22.02)	.	.
3	-11.90 (-20.18; -3.62)	12.15 (-9.79; 34.08)	A1B + IBB	.	.	.	.	.	.	.	.	.	.	.	.	.
4	-18.86 (-31.55; -6.18)	5.18 (-12.18; 22.54)	-6.96 (-22.11; 8.18)	AB	-0.20 (-13.75; 13.35)	12.56 (3.89; 21.23)	.	.	-6.70 (-19.30; 5.90)	-0.66 (-11.14; 9.81)	5.90 (-1.77; 13.57)	.	5.60 (-2.01; 13.21)	1.40 (-6.57; 9.37)	.	.
5	-12.63 (-26.54; 1.29)	11.42 (-7.28; 30.12)	-0.73 (-16.92; 15.46)	6.24 (-2.01; 14.49)	B-TCP	3.39 (-5.40; 12.19)	-2.20 (-13.04; 8.64)	.	.	.	.	.	.	.	.	.
6	-8.15 (-22.43; 6.14)	15.90 (-2.86; 34.67)	3.75 (-12.75; 20.26)	10.72 (2.92; 18.52)	4.48 (-3.30; 12.26)	B-TCP + AB	.	.	.	.	.	.	.	.	.	.
7	-14.51 (-26.66; -2.35)	9.54 (-8.40; 27.48)	-2.61 (-17.31; 12.10)	4.36 (-3.60; 12.32)	-1.88 (-10.40; 6.65)	-6.36 (-16.09; 3.37)	BCP	.	.	.	.	.	0.51 (-5.43; 6.45)	.	.	.
8	-12.22 (-27.22; 2.78)	11.82 (-8.29; 31.93)	-0.32 (-17.45; 16.81)	6.64 (-5.72; 19.00)	0.40 (-13.21; 14.02)	-4.08 (-18.07; 9.91)	2.28 (-9.53; 14.10)	BCP + PRF	.	.	.	.	.	.	.	-3.30 (-11.59; 4.99)
9	-27.05 (-44.14; -9.96)	-3.00 (-23.79; 17.79)	-15.15 (-34.13; 3.84)	-8.18 (-19.63; 3.26)	-14.42 (-28.53; -0.31)	-18.90 (-32.75; -5.05)	-12.54 (-26.48; 1.40)	-14.82 (-31.67; 2.02)	Bioglass	8.80 (-3.19; 20.79)	.	.	.	.	.	.

10	-19.53 (-35.98; -3.08)	4.52 (-15.76; 24.79)	-7.63 (-26.04; 10.79)	-0.66 (-11.14; 9.81)	-6.90 (-20.23; 6.43)	-11.38 (-24.44; 1.67)	-5.02 (-18.18; 8.13)	-7.31 (-23.51; 8.89)	7.52 (-3.57; 18.61)	Bioglass + AB	.	.	.	.	.	.
11	-12.55 (-25.00; -0.11)	11.49 (-5.78; 28.77)	-0.65 (-15.60; 14.29)	6.31 (-0.80; 13.42)	0.07 (-9.73; 9.87)	-4.41 (-14.41; 5.59)	1.95 (-6.11; 10.02)	-0.33 (-12.44; 11.78)	14.49 (1.02; 27.97)	6.97 (-5.68; 19.63)	HA	-5.00 (-15.18; 5.18)	-1.29 (-7.58; 4.99)	-4.50 (-12.37; 3.37)	.	.
12	-17.55 (-33.63; -1.48)	6.49 (-13.56; 26.54)	-5.65 (-23.74; 12.43)	1.31 (-11.11; 13.73)	-4.93 (-19.06; 9.20)	-9.41 (-23.68; 4.86)	-3.05 (-16.04; 9.94)	-5.33 (-21.15; 10.49)	9.49 (-7.39; 26.38)	1.97 (-14.27; 18.22)	-5.00 (-15.18; 5.18)	HA + PRF	.	.	.	.
13	-13.90 (-24.70; -3.10)	10.15 (-7.06; 27.35)	-2.00 (-15.60; 11.60)	4.96 (-1.70; 11.63)	-1.27 (-10.05; 7.50)	-5.75 (-15.10; 3.59)	0.61 (-4.98; 6.19)	-1.68 (-12.09; 8.74)	13.15 (-0.10; 26.39)	5.63 (-6.78; 18.04)	-1.35 (-7.53; 4.84)	3.65 (-8.26; 15.56)	IBB	-4.20 (-12.02; 3.62)	-6.94 (-12.66; -1.22)	-1.62 (-7.92; 4.67)
14	-17.55 (-30.65; -4.44)	6.50 (-9.02; 22.02)	-5.65 (-21.15; 9.85)	1.32 (-6.46; 9.10)	-4.92 (-15.35; 5.51)	-9.40 (-19.95; 1.15)	-3.04 (-12.04; 5.96)	-5.32 (-18.11; 7.47)	9.50 (-4.34; 23.34)	1.98 (-11.06; 15.03)	-4.99 (-12.58; 2.59)	0.01 (-12.69; 12.70)	-3.65 (-11.07; 3.78)	IBB + AB	.	.
15	-20.84 (-33.06; -8.62)	3.21 (-14.93; 21.34)	-8.94 (-23.70; 5.82)	-1.98 (-10.76; 6.81)	-8.21 (-18.69; 2.26)	-12.70 (-23.66; -1.74)	-6.34 (-14.33; 1.66)	-8.62 (-20.50; 3.26)	6.21 (-8.22; 20.63)	-1.31 (-14.98; 12.35)	-8.29 (-16.71; 0.14)	-3.29 (-16.50; 9.93)	-6.94 (-12.66; -1.22)	-3.29 (-12.67; 6.08)	IBB + BMC	.
16	-15.52 (-28.02; -3.02)	8.52 (-9.80; 26.84)	-3.62 (-18.61; 11.37)	3.34 (-5.83; 12.51)	-2.90 (-13.70; 7.90)	-7.38 (-18.65; 3.89)	-1.02 (-9.44; 7.40)	-3.30 (-11.59; 4.99)	11.52 (-3.14; 26.19)	4.01 (-9.91; 17.92)	-2.97 (-11.79; 5.86)	2.03 (-11.44; 15.50)	-1.62 (-7.92; 4.67)	2.02 (-7.71; 11.76)	5.32 (-3.19; 13.82)	IBB + PRF

Table S2. P-score values ranging from highest (poor efficiency) to lowest (better efficiency) compared to AB.

Intervention	P-score
A1B	0.9788
B-TCP+AB	0.8423
HA	0.6782
BCP+PRF	0.6588
B-TCP	0.6489
A1B+IBB	0.6420
IBB	0.6016
BCP	0.5529
IBB+PRF	0.4872
HA+PRF	0.3999
IBB+AB	0.3824
Bioglass+AB	0.3224
AB	0.3030
IBB+BMC	0.2150
A1B+AB	0.2028
Bioglass	0.0839

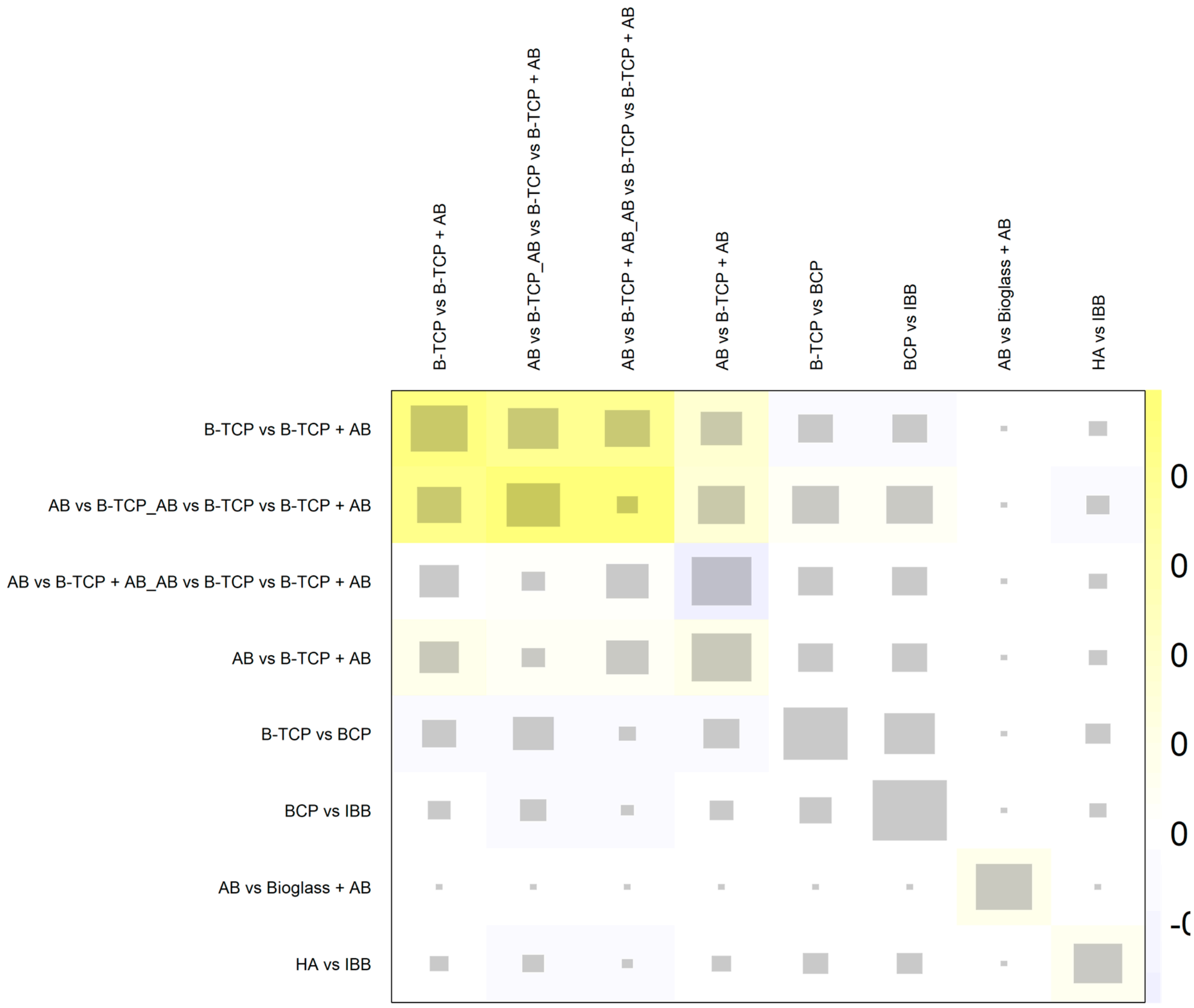

Figure S2. Heat plot for NBRR.
